# Transcriptome Analysis of the Sydney Rock Oyster, *Saccostrea glomerata*: Insights into Molluscan Immunity

**DOI:** 10.1371/journal.pone.0156649

**Published:** 2016-06-03

**Authors:** Nicole G. Ertl, Wayne A. O’Connor, Alexie Papanicolaou, Aaron N. Wiegand, Abigail Elizur

**Affiliations:** 1 University of the Sunshine Coast, Sippy Downs, Queensland, Australia; 2 Australian Seafood Cooperative Research Centre, Bedford Park, South Australia, Australia; 3 Department of Primary Industries, Taylors Beach, New South Wales, Australia; 4 Commonwealth Scientific and Industrial Research Organisation (CSIRO) Ecosystem Sciences, Black Mountain Laboratories, Canberra, Australian Capital Territory, Australia; Institute of Oceanology, Chinese Academy of Sciences, CHINA

## Abstract

**Background:**

Oysters have important ecological functions in their natural environment, acting as global carbon sinks and improving water quality by removing excess nutrients from the water column. During their life-time oysters are exposed to a variety of pathogens that can cause severe mortality in a range of oyster species. Environmental stressors encountered in their habitat can increase the susceptibility of oysters to these pathogens and in general have been shown to impact on oyster immunity, making immune parameters expressed in these marine animals an important research topic.

**Results:**

Paired-end Illumina high throughput sequencing of six *S*. *glomerata* tissues exposed to different environmental stressors resulted in a total of 484,121,702 paired-end reads. When reads and assembled transcripts were compared to the *C*. *gigas* genome, an overall low level of similarity at the nucleotide level, but a relatively high similarity at the protein level was observed. Examination of the tissue expression pattern showed that some transcripts coding for cathepsins, heat shock proteins and antioxidant proteins were exclusively expressed in the haemolymph of *S*. *glomerata*, suggesting a role in innate immunity. Furthermore, analysis of the *S*. *glomerata* ORFs showed a wide range of genes potentially involved in innate immunity, from pattern recognition receptors, components of the Toll-like signalling and apoptosis pathways to a complex antioxidant defence mechanism.

**Conclusions:**

This is the first large scale RNA-Seq study carried out in *S*. *glomerata*, showing the complex network of innate immune components that exist in this species. The results confirmed that many of the innate immune system components observed in mammals are also conserved in oysters; however, some, such as the TLR adaptors MAL, TRIF and TRAM are either missing or have been modified significantly. The components identified in this study could help explain the oysters’ natural resilience against pathogenic microorganisms encountered in their natural environment.

## Background

Oysters are an important ecological invertebrate species, carrying out a wide range of environmental functions. For instance, oysters filter nutrients, suspended solids and phytoplankton from the water column which lowers turbidity and increases water quality. Furthermore, they act as global carbon sinks, sequestering carbon from the ocean and depositing it in their shell matrix as calcium carbonate. Oysters and oyster reefs also protect shorelines and salt marshes from wave erosion, influence sediment distribution and can provide a habitat for other benthic and epibenthic species [1–5]. In addition to their ecological value, oysters such as the iconic Sydney rock oyster (*Saccostrea glomerata*) also have a substantial economic value, having contributed about AU$30 million to the New South Wales (Australia) economy in 2012/2013 [6]. In their natural habitat, oysters are exposed to a range of pathogens that can cause mass mortalities. Some of these pathogens are *Haplosporidium nelsoni* and *Perkinsus marinus* that cause MSX and Dermo, respectively in *Crassostrea virginica* Gmelin, with mortality rates of infected oysters between 50–90% [7]. Mortalities in bivalve hatcheries have been attributed to bacteria of the genus *Aeromonas*, *Pseudomonas*, *Vibrio* and *Nocardia* [8], and *Ostreid herpesvirus 1* (OsHV-1) is the cause for repeated mass mortalities in *Crassostrea gigas*, with a variant of the virus associated with mortalities in *Ruditapes phillipinarum*, *C*. *gigas* and *Pecten maximus* [9]. The paramyxean protozoan of the genus *Marteilia* has also been shown to cause mass mortalities in several oyster species. For example, *Marteilia refringens* (Aber disease) was implicated in mass mortalities in *Ostrea edulis* and *Marteilioides chungmuensis* appears to be a pathogen of *C*. *gigas* [10]. *Marteilia sydneyi* is known to cause Queensland unknown (QX) disease in Sydney rock oysters, with mortality rates of up to 98% during an outbreak [11, 12]. While breeding of QX survivors has shown improvement in their ability to withstand QX disease, mortality in this breeding line was observed to increase during second season exposure to QX [13]. Previous studies have shown that environmental stressors (e.g. reduced salinity, pollution) to which oysters are exposed to in their natural habitat, can have detrimental effects on their immune functions. These in turn can increase their susceptibility to diseases such as QX or Dermo [14–17]. For instance, Cherkasov *et al*. [18] observed a significant increase in circulating haemocyte mortality in *C*. *virginica* exposed to elevated temperature and Kuchel *et al*. [19] reported a significant decrease in phagocytosis in response to air exposure, low salinity and mechanical stress. Considering the ecological and economical importance of oysters and their exposure to environmental stress and pathogens in their natural habitat, a strong innate immunity is essential for oyster health and survival. Furthermore, a better understanding of the molecular basics of the immune system in oysters and other molluscs is needed to develop strategies that increase oyster resilience to environmental stressors and diseases.

Next generation sequencing (NGS) provides large sequencing datasets at a fast sequencing speed and more lately at an affordable price [20]. This technology has already been used to produce two oyster genomes, the genome of *C*. *gigas* [21] and the draft genome of *Pinctada fucata* [22]. Furthermore, NGS provided insights into a range of biological functions in molluscs such as biomineralisation and immunity in *Pinctada martensii* [23], biomineralisation in *Pinctada margaritifera* [24], immunity in *C*. *virginica* and *Mytilus edulis* [25, 26] and sex differentiation in *P*. *margaritifera* [27]. While immunity has been assessed in some molluscs, it has not yet been assessed in the iconic Sydney rock oysters for which only limited sequencing information is publically available [28]. Therefore, in this study, we exposed *S*. *glomerata* to a range of environmental stressors and sequenced six tissues of stressed and non-stressed adult oysters (samples pooled per tissue type) to obtain a broad spectrum of genes expressed in this species. Resulting *S*. *glomerata* raw Illumina sequencing reads were cleaned, assembled and open reading frames (ORFs) analysed for potential immune and immune related genes. Furthermore, transcript expression patterns across the different tissues were examined. The results of the analysis are presented in this study.

## Results and Discussion

### *S*. *glomerata* transcriptome sequencing and assembly

In order to capture a broad spectrum of genes actively expressed in *S*. *glomerata* in response to stress, adult oysters were exposed to different potential stressors (CO_2_, salinity, temperature, copper and polycyclic aromatic hydrocarbons) and tissue samples (haemolymph, gill, mantle, adductor muscle, gonad and digestive) extracted at multiple sampling time-points (treatment details are presented in S1 File). Normalised strand-specific libraries, as well as non-normalised and non-strand specific libraries were prepared from each of the tissues and sequenced using the Illumina technology, resulting in a total of 484,121,702 paired-end reads with a GC content of 44–46%. Similar GC contents have been found in other molluscs, for example in the snail *Bythinia siamensis goniomphalos* (44.4%) and the oyster *Pinctada maxima* (43.2%) [29, 30]. Of the raw reads, 99.7% were retained past quality control and pre-processing and then assembled into contigs with Trinity RNASeq [31]. Two reference transcriptomes were produced: one derived only from the strand-specific data (strand-specific transcriptome) and one derived from all the data (combined transcriptome). Assembly statistics before and after redundancy removal are summarised in Table 1. As expected due to increased coverage, the combined transcriptome assembly had 20.7% more ORF predictions, a higher N50 value and longer transcripts. However, the increased coverage may have caused issues due to the high degree of polymorphism in this species. When assessing completeness using the CEGMA approach [32], more core eukaryotic genes were found to be present in the strand-specific than the combined transcriptome (Table 1). While the CEGMA software was originally developed to assess genomic assemblies for completeness, it has also been used to assess transcriptomes [33, 34]. In addition to the CEGMA analysis, the N50 values obtained for the *S*. *glomerata* transcriptomes were similar to the N50 values of other molluscan transcriptomes [25, 35, 36]. Based on these results, both assemblies were considered to be of a suitable quality for further analysis and therefore used in this study.

**Table 1:** Summary of *S*. *glomerata* assembly, ORF prediction and CEGMA analysis.

	Strand-specific transcriptome	Combined transcriptome
**Total transcripts (#)**	519,639	502,645
N50 length (bp)	949	1,238
Mean transcript length (bp)	633	701
Min transcript length (bp)	201	201
Max transcript length (bp)	27,590	30,775
**Non-redundant transcripts (#)**	414,578	453,096
N50 length (bp)	768	937
Mean transcript length (bp)	564	613
Min transcript length (bp)	201	201
Max transcript length (bp)	27,590	30,775
*n* transcripts < 500 bp	292,626	318,450
*n* transcripts 500–1000 bp	72,056	73,425
*n* transcripts > 1000 bp	49,896	61,221
**ORF predictions (#)**	85,786	108,130
Min length (bp)	300	300
Max length (bp)	25,917	29,883
**CEGMA**		
Complete proteins (%)	95.56	91.94
Partial proteins (%)	99.19	95.56

### *C*. *gigas* comparison and transcript annotation

Reads and reference transcriptomes from *S*. *glomerata* were aligned to the *C*. *gigas* genome to examine the level of similarity of the two oyster species. Strand-specific and normalised reads, as well as the strand-specific reference transcriptome showed higher mapping rates to the *C*. *gigas* genome than the non-normalised and non-strand specific reads and combined transcriptome (21.4% and 3.4% versus 15.6% and 2.1%, respectively). The observed low transcript alignment percentages (3.4% and 2.1%) indicate that the majority of *S*. *glomerata* transcripts overall have less than 60% similarity to the *C*. *gigas* genome at the nucleotide level. However, at the deduced protein level *C*. *gigas* was the closest match, with 81.2% and 75.7% of the best blast matches for the *S*. *glomerata* strand-specific and combined ORF’s, respectively being to protein sequences of *C*. *gigas*. Other molluscan best-hit matches were with *Lottia gigantea*, *Aplysia californica*, *Ostrea edulis*, *Mytilus galloprovincialis* and *Crassostrea ariakensis* (S1a and S1b Fig). Similar to our study, Zhang *et al*. [25] mapped *C*. *virginica* Illumina paired-end reads to the *C*. *gigas* genome, showing that only 3.43% of raw reads aligned to the genome, while over 99% of *C*. *virginica* contigs could be annotated by the *C*. *gigas* protein set, thus also showing a much higher similarity at the protein rather than nucleotide level.

Functional annotation was carried out on the 85,786 strand-specific and 108,130 combined *S*. *glomerata* ORFs, using Blast2GO. A total of 83.5% and 81.4% of the strand-specific and combined ORFs, respectively were annotated with NCBI’s non-redundant database, using an e-value cut-off of 1e^-5^. When all ORFs were searched against the InterProScan database, 80.1% and 79.6% of strand-specific and combined ORFs, respectively could be annotated. Of the functionally annotated ORFs for both transcriptomes, GO-terms associated with metabolic and cellular processes, binding and catalytic activity, and cells and membranes contained the most ORFs (S2 and S3 Figs). Similar GO-terms have been found in other molluscs such as *C*. *virginica* and the clam *Meretrix meretrix* [25, 37]. In addition, both *S*. *glomerata* transcriptomes had a range of GO-terms associated with responses to different stimuli/stress (e.g. response to chemical stimulus, detection of stimulus) and to immunity (e.g. immune response, death) (S2a and S3a Figs).

### Tissue distribution of transcripts

**Fig 1:**
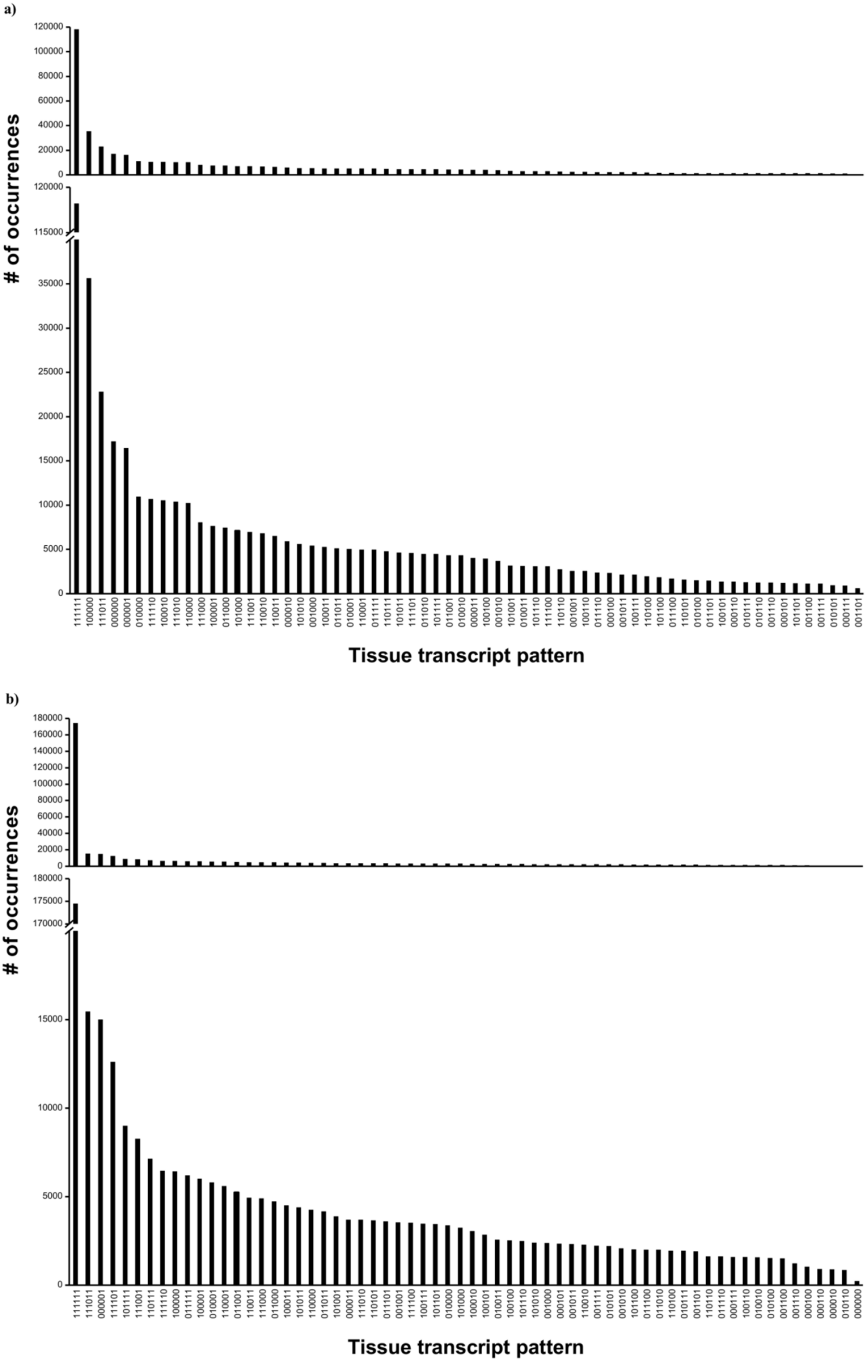
Transcript patterns across six different *S*. *glomerata* tissues. **A)** non-normalised and non-strand specific tissue reads were mapped to the combined reference transcriptome and **b)** strand-specific and normalised tissue reads were mapped to the strand-specific reference transcriptome, using CLC Workbench version 7.5. Graph shows the number of transcripts that are common across individual tissue patterns, with 1 standing for “transcript present in tissue” and 0 for “transcript not present in tissue”. The order in which the tissues are listed is as follows: haemolymph (closest to the x-axis label), gill, mantle, muscle, gonad and digestive. The respective top graphs show the full-scale graph, with the respective lower graphs emphasising the fine scale features of the same graph by adding a graph break.

*S*. *glomerata* reads (S1 Table) were mapped to their respective reference transcriptome and the transcript expression pattern across the six tissues examined. The largest number of *S*. *glomerata* transcripts (42.1% and 26.1% for strand-specific and combined, respectively) were found to be expressed in all tissues (Fig 1a and 1b). As most eukaryotic cells express the same set of cell homeostasis related genes [38], it was expected to see a high number of transcripts expressed in all *S*. *glomerata* tissues tested, with a smaller amount of transcripts specifically expressed in certain cells and tissues depending on the function of these cells and tissues. This has been observed in *S*. *glomerata*, where transcripts expressed in all tissues but the adductor muscle, transcripts only expressed in the digestive system and transcripts only expressed in the haemolymph (Fig 1a and 1b) were among the ten highest tissue patterns found in the *S*. *glomerata* transcriptomes. Some of the transcripts found to be expressed only in the haemolymph coded for cathepsins (e.g. specific transcripts coding for cathepsin B and L), heat shock proteins (e.g. specific transcripts coding for heat shock protein 70 and 90), peroxiredoxin (e.g. specific transcripts coding for peroxiredoxin 6) and superoxide dismutase (e.g. some transcripts with mitochondrial manganese-superoxide dismutase domains). Of these transcripts, peroxiredoxin and superoxide dismutase have been shown to act as antioxidants [39, 40], while cathepsins are lysosomal proteolytic enzymes involved in phagocytosis [41]. Heat shock proteins, on the other hand, are involved in the response to stress and have also been shown to act as endogenous ligands for Toll-like receptors (TLRs) in mammals, which activate the TLR signalling pathway, resulting in inflammatory responses [42, 43].

### Immune and immune-related genes

Innate immunity is the first and only line of defence against invading pathogens in invertebrates such as oysters. Considering that aquatic habitats harbour a range of bacteria and viruses (approximately 10^29^ and 10^10^ cells/L of prokaryotes and viruses, respectively), aquatic animals such as oysters that inhabit this environment strongly rely on the protective functions of the innate immune system [44]. The innate immune response contains cellular (e.g. phagocytes) and humoral (e.g. enzymes) components that protect the bivalve host from harmful microorganisms that it encounters in its natural environment [45, 46]. In general terms, immune cells (e.g. haemocytes) are activated by the stimulation of receptors by pathogen-associated molecular patterns (PAMPs). This immune response involves signalling cascades and the release/production of various molecules such as hydrolytic enzymes and antimicrobial peptides that are part of the immune arsenal against pathogens [47]. In the following sections we describe the different components identified in this transcriptome study that are potentially involved in innate immunity in the Sydney rock oyster.

### Pattern recognition receptors (PRRs)

Through interactions with their environment, oysters are continuously exposed to a broad range of microorganisms. Different to vertebrates, oysters lack an adaptive immune system and thus have to rely heavily on the innate immune system as protection from invading microorganisms [48]. PRRs are an important part of the innate immune defence as they are able to recognise PAMPs (e.g. lipopolysaccharide [LPS], bacterial DNA and viral RNA) and endogenous ligands (e.g. heat shock proteins), also termed damage-associated molecular patterns (DAMPs), that are generally released in response to tissue injury or cell stress [42, 44, 49, 50]. Stimulation of PRRs induces intracellular signalling pathways, leading to inflammatory immune responses. In addition, PRRs are also linked to phagocytosis and the complement pathway [42, 49]. Similar to vertebrates, several PRRs have been discovered in invertebrates such as crustaceans and molluscs. These include, for instance, Toll-like receptors (TLRs) and Down syndrome cell adhesion molecules (DSCAM) [43, 51]. Akin to the PRRs found in other invertebrates, a range of PRRs has also been detected in the *S*. *glomerata* transcriptomes of this study. Multiple transcripts of TLRs, peptidoglycan recognition proteins (PGRPs), gram-negative bacteria binding proteins (GNBPs), c-type lectins, collectins, ficolins, macrophage mannose receptors, galectins, RIG-I-like receptors (RLRs), thioester-containing proteins (TEPs), fibrinogen-related proteins (FREPs), scavenger receptors (SRs), as well as DSCAM were found to be expressed in the *S*. *glomerata* transcriptomes (Table 2). TLRs, which are a key component of the immune system, have been studied over many years and appear to be conserved across vertebrates and invertebrates alike and are expressed in mammals, flies, crustaceans and molluscs [43, 51, 52]. Along with the TLRs found in our study, TLR sequences have also been observed in other molluscs such as the mussels *Bathymodiolus azoricus* and *M*. *edulis*, the scallop *Pecten maximus* and the oyster *C*. *virginica* [25, 26, 53, 54]. Furthermore, most of the other PRRs detected in our study have also been reported in *C*. *virginica*, *M*. *edulis* and *P*. *maximus*, from SRs, PGRPs, GNBPs, c-type lectin and galectin to collectins and TEPs [25, 26, 53]. Contradictory to the observation of Zhang *et al*. [25], ficolins, which have been linked to the complement system [55], have already been detected in molluscs, with transcripts found in the scallop *P*. *maximus* [53] and the *C*. *gigas* genome [21], as well as in our *S*. *glomerata* transcriptomes. Xiang *et al*. [55] showed an increased expression of a ficolin-like gene in *Crassostrea hongkongensis* after the oyster was challenged with microbes, with the recombinant protein being able to agglutinate *Escherichia coli* K-12 in the presence of Ca^2+^ and raising the phagocytic activity of *C*. *hongkongensis* haemocytes. It is conceivable that the *S*. *glomerata* ficolins found in our study could have a similar functionality as the ficolin-like in *C*. *hongkongensis*, which would allow *S*. *glomerata* to not only recognise invading pathogens but to also act as opsonins, increasing the likelihood that the pathogens will be cleared from the host by phagocytosis.

**Table 2:** Potential *S*. *glomerata* immune and immune related ORFs determined by GO and/or InterProScan annotation.

	# of ORFs	Details
**Pattern recognition receptors (PRRs)**		
peptidoglycan recognition proteins (PGRPs)	23	
gram-negative bacteria binding proteins (GNBPs)	3	
Collectins	53	
Ficolins	30	
c-type lectins	110	
macrophage mannose receptors	237	
scavenger receptors (SRs)	26	based on domain information in [56]
thioester-containing proteins (TEPs)	31	
fibrinogen-related proteins (FREPs)	40	
down syndrome cell adhesion molecule (DSCAM)	11	
Galectins	20	
Toll-like receptors (TLRs)	220	
interferon-induced helicase c domain-containing protein 1 (MDA5)/(also RIG-I called) DDX58	22	
stabilin-2	3	
**Immune signaling pathways**		
myeloid differentiation primary response protein 88 (MyD88)	14	
sterile alpha and TIR domain containing protein (SARM1)	3	
IL-1R-associated kinase (IRAK)	1	
tumor necrosis factor [TNF] receptor-associated factor (TRAF)	31	TRAF7 (8), TRAF6 (3), TRAF2 (3), TRAF4 (3), TRAF3 (1), TRAF6-like isoform 1 (1)
inhibitor of nuclear factor kappa-b kinase (IKK)	6	
transforming-growth-factor [TGF]-β-activated kinase 1 (TAK1, also called MEKK7)	6	
TAK1-binding protein (TAB1)	3	
TAK1-binding protein 2 (TAB2)	2	
nuclear factor [NF]-κB essential modulator (NEMO)	2	
TRAF-family-member-associated NF-κB activator-binding kinase 1 (TBK1)	13	
inhibitor of nuclear factor-κB (IκB)	4	
nuclear factor of kappa light polypeptide gene enhancer in b-cells 2, p49/p100 (NFκB2)	2	
activator protein 1 (AP-1)	1	
interferon regulatory factor (IRF)	7	
janus activated kinase (JAK)	2	
signal transducer and activator of transcription (STAT)	4	
mitogen-activated protein kinases (MAPK)	13	MAPK3 (1), MAPK14 (4), MAPK15 (2), MAPK6 (3) and MAPK7 (1)
mitogen-activated protein kinase kinase (MEK also called MKK)	11	
mitogen-activated protein kinase kinase kinase (MAP3K or MEKK)	34	MEKK1 (2), MEKK19 (8), MEKK2 (1), MEKK10 (1), MEKK15 (1), MEKK9 (1), MEKK13 (2)
mitogen-activated protein kinase kinase kinase MLT-like isoform 1 (also called ZAK isoform 1)	1	
mitogen-activated protein kinase kinase kinase kinase (MAP4K)	10	MAP4K3 (6) and MAP4K5 (4)
receptor-interacting protein 1 (RIP1)	3	
Pellino	2	
toll-interacting protein (TOLLIP)	1	
suppressor of cytokine signaling (SOCS)	5	
NF-kappa-B inhibitor-like protein 1 (NFKBIL1)	1	
interleukin-1 receptor-associated kinase 1-binding protein 1 (IRAK1BP1)	1	
mitochondrial antiviral-signaling protein (MAVS)	1	
nuclear factor of activated T-cells (NFAT)	1	
c-jun-amino terminal kinase-interacting protein 4 (JIP4)	12	
c-jun-amino terminal kinase-interacting protein 1 (JIP1)	1	
*endogenous ligands*		
fibrinogen (α/β/γ-chain)	8	
heat shock protein [Hsp]20 family	13	
Hsp70 family	40	
Hsp90 family	25	
Hsp60	2	
Hsp10	1	
stress-induced-phosphoprotein 1 (STIP1)	2	
intraflagellar transport protein 25 homolog (Hspb11)	2	
*Hypoxia-inducible factors (HIFs)*		
HIF-1α / HIF-2α	6	
HIF-1β	2	
hypoxia-inducible factor 1-α inhibitor (HIF1AN)	1	
prolyl hydorxylases (PHD)	2	
p300 / CREB-binding protein (CBP)	13	
**Immune effectors**		
*Antioxidant defence system/phagocytosis*		
superoxide dismutase (SOD)	25	with mitochondrial manganese-SOD domains (4) and copper/zinc-SOD domains (21)
catalase (CAT)	7	
glutathione peroxidase (GPX)	7	
nitric oxide synthase (NOS)	2	
Thioredoxin	6	
thioredoxin reductase	4	
Peroxiredoxin	11	
Glutaredoxin	6	
glutathione S-transferase (GST)	53	
glutathione synthase (GS)	2	
glutamate—cysteine ligase regulatory subunit	1	
glutamate—cysteine ligase catalytic subunit	3	
methionine sulfoxide reductase A	3	
methionine-R-sulfoxide reductase B3	4	
glutathione reductase (mitochondrial)	1	
NADPH oxidase (NOX)	4	
p67^phox^	1	
cytochrome b-245 heavy chain-like (gp91^phox^)	1	
cytochrome b-245 light chain-like (p22^phox^)	1	
dual oxidase (DUOX)	7	
small GTPase Rac	2	
evolutionarily conserved signaling intermediate in toll pathways, mitochondrial (ECSIT)	1	
Cathepsin	59	cathepsin B (9), F (3), L (17), C (1), O (2), Z (1), D (3)
Lysozymes	16	
*Antimicrobial peptides*		
big defensins	5	
Hydramacin	1	
bactericidal permeability increasing protein (BPI)	27	
*cytokines and cytokine receptors*		
macrophage migration inhibitory factor (MIF)	3	
interleukin [IL]-17	3	
interleukin-17 receptor d	5	
interleukin-6 receptor	12	
*Others*		
Septin	7	
c4b-binding protein alpha/beta chain	8	
**Apoptosis**		
FAS-associated death domain protein (FADD)	3	
TNF receptor superfamily members	9	
TNF superfamily members	8	
apoptosis-inducing factor 1, mitochondrial (AIF)	1	
Bax	2	
Caspases	114	
baculoviral IAP repeat-containing proteins (IAPs)	75	
direct IAP-binding protein of low isoelectric point (DIABLO)	4	
interferon alpha-inducible protein 27 (IFI27)	25	

RLRs (MDA5 and RIG-I), another important PRR group aside from TLRs were first observed in *M*. *edulis* [26]. RLRs are important sensors for viruses in the cytoplasm, with downstream immune signalling initiated after virus recognition [26, 49]. These PRRs have also been detected in the *S*. *glomerata* transcriptomes of our study (Table 2), suggesting that the immune system of *S*. *glomerata* might be able to defend the oyster from intracellular viruses. One other family of PRR found in mammals aside from TLRs are NOD-like receptors (NLRs) that are able to recognise and elicit an immune response to bacterial motifs in the cytoplasm [49]. While sequence homology searches revealed matches to NLRs in our *S*. *glomerata* transcriptomes, these matches could not be confirmed with InterProScan and comparison to curated NLR sequences on uniprot. Similar to our study, Philipp *et al*. [26] also did not find any NLRs in their *M*. *edulis* transcriptome, indicating that in contrast to TLRs and RLRs, NLRs might not be conserved in bivalves.

**Fig 2:**
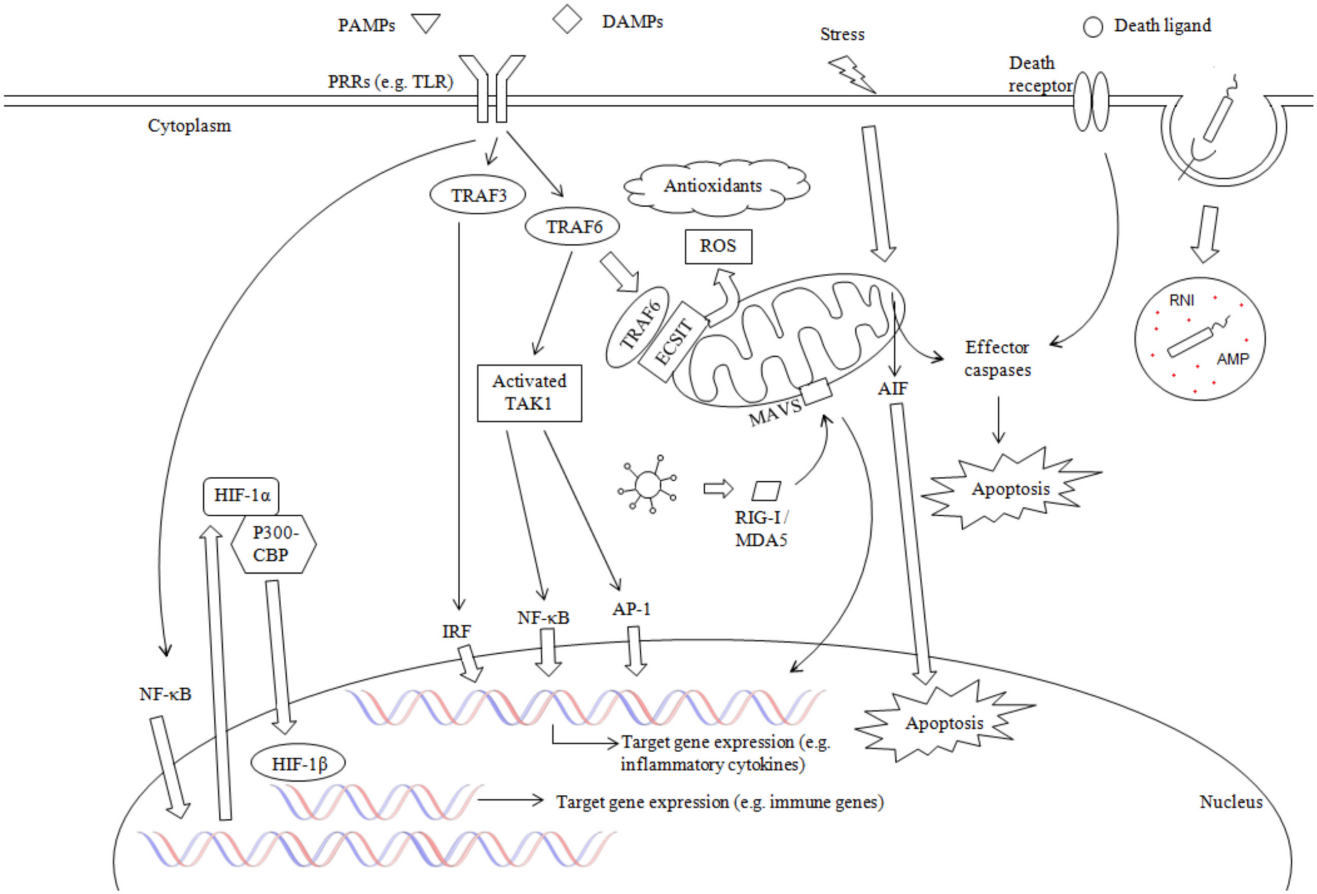
Schematic of the predicted innate immune processes of *S*. *glomerata*. *S*. *glomerata* innate immune transcripts observed in this study are predicted to be involved in a range of different innate immune processes, (components regulating the different pathways are not visualised in this figure). Abbreviations are as follows: PPRs (pattern recognition receptors), TLR (Toll-like receptor), PAMPs (pathogen-associated molecular patterns), DAMPs (damage-associated molecular patterns), TRAF (tumor necrosis factor receptor-associated factor), TAK1 (transforming-growth-factor-β-activated kinase 1), ROS (reactive oxygen species), ECSIT (evolutionary conserved signalling intermediate in toll pathways), RIG-1/MDA5 (interferon-induced helicase c domain-containing protein 1), MAVS (mitochondrial antiviral-signalling protein), AIF (apoptosis-inducing factor 1), AP-1 (activator protein 1), NF-κB (nuclear factor kappa B), IRF (interferon regulatory factor), HIF (hypoxia-inducible factor), CBP (CREB-binding protein), RNI (reactive nitrogen intermediates), AMP (antimicrobial peptide). ↑ arrows used in the figure (excluding the ones used in the nucleus) depict signalling pathways.

Some of the PRRs found in invertebrates have also been examined for their involvement in innate immunity. For instance, SR expression in the scallop *Chlamys farreri* was induced in response to PAMPs (LPS, PGN and β-glucan), with the SR recombinant protein able to bind LPS, PGN and the fungal particles mannan and zymosan in the presence of Ca^2+^ [56]. Other PRRs examined were TEP, FREPs and DSCAM, which were observed to respond to bacteria in invertebrates, with DSCAM also associated with phagocytosis [43, 51]. These results indicate that PRRs in invertebrates also function in PAMPs detection, as their mammalian counterparts do. Of the PRRs found in invertebrates and the *S*. *glomerata* transcriptome of this study, members of the c-type lectin superfamily have also been linked to the complement system in vertebrates [57–59]. This family consists of c-type lectins, collectins and macrophage mannose receptor, with the latter associated with the cell membrane and linked to phagocytosis [57]. Ficolins on the other hand are serum proteins (except M-ficolin) that have been shown to activate the complement pathway [58, 59]. Having a range of PRRs that act in different compartments (e.g. haemolymph), respond to a wide range of pathogens and activate different pathways (e.g. TLR signalling pathway) might give *S*. *glomerata* an edge against invading pathogens, especially as the innate immune system is the only defence system oysters possess, making it essential to have a broad range of PAMPs recognition molecules to activate downstream defence mechanisms (Fig 2).

### TLR signalling pathway

TLRs are well characterised and have been found in both vertebrates and invertebrates. They have a key role in the recognition of microbes and their stimulation by a range of exogenous and endogenous ligands (e.g. double-stranded viral RNA, LPS, HSP60 and HSP70) leads to the activation of a highly conserved signalling pathway [26, 42, 44, 51, 60]. Once the ligand interacts with its respective TLR, the receptor is activated, after which the TLR signalling domain dimerises and recruits adaptors [42, 50]. Five adaptor proteins have been found so far, myeloid differentiation primary-response protein 88 (MyD88), MyD88-adaptor-like protein (MAL, also called TIRAP), toll/interleukin-1 receptor (TIR) domain-containing adaptor protein inducing interferon [IFN]-β (TRIF or TICAM1), TRIF-related adaptor molecule (TRAM, also termed TIRP) and sterile-α-and armadillo-motif-containing protein 1 (SARM1) [42, 49, 50]. While the first four adaptor proteins lead to downstream signalling, SARM1 has been found to inhibit TRIF [52]. Of the five adaptor proteins, only MyD88 and SARM1 were found in the *S*. *glomerata* transcriptome along with multiple ORFs coding for TLRs (Table 2). Even though MAL appears to be important in mammalian TLR signalling, this adaptor, as well as TRIF and TRAM have not been found in *S*. *glomerata*, which overall appears to be consistent with results in *M*. *edulis* and *Strongylocentrotus purpuratus* where distinct orthologues of TRAM and TRIF could not be found [26, 61]. Furthermore, as MAL, TRIF and TRAM have only been detected in chordates so far [62], it seems that neither of these adaptors might be used in molluscs. Although SARM1 acts as an inhibitor in the mammalian TLR signalling pathway, contradictory functions of SARM1 have been observed in horseshoe crabs and *Caenorhabditis elegans* [63]. In the crustacean, SARM1 functioned as inhibitor, whereas in *C*. *elegans* its function resulted in the production of antimicrobial peptides [63], suggesting that SARM1 could potentially act as a second adaptor next to MyD88 in *S*. *glomerata*, leading to downstream signalling and the induction of the innate immune system. However, further studies are needed to clarify the exact function(s) of SARM1 in invertebrates and especially in molluscs. Research into the expression levels of nine TLRs and three MyD88s in *M*. *edulis* challenged with the bacteria *Vibrio splendidus*, *V*. *anguillarum* and *Micrococcus luteus* and the fungus *Fusarium oxysporum*, showed that one TLR responded to the bacteria and fungus with an increase in gene expression [64]. In addition, all three MyD88 had increased levels of expression after challenge with bacteria, with only one of the MyD88 also stimulated by the fungus [64]. A similar pattern was shown in *M*. *galloprovincialis*, where bacterial challenge resulted in an increase in expression levels in one out of five TLRs and all three tested MyD88s [65]. These results indicate that both TLRs and the adaptor protein MyD88 respond to a range of pathogens and play an important role in the innate immunity of bivalves such as *S*. *glomerata*.

In mammals, the specific adaptor(s) recruited to the individual TLR determines the downstream signalling pathway, activating either the MyD88-dependent or the TRIF-dependent pathway. All TLRs but TLR3 have been shown to utilise the MyD88-dependent pathway that eventually results in the production of inflammatory cytokines. TLR3 on the other hand uses the TRIF-dependent pathway that leads to the production of inflammatory cytokines as well as the expression of type I IFNs [49, 50]. Different to most TLRs, TLR4 has been shown to be able to initiate both, the MyD88-dependent and the TRIF-dependent pathway [49, 50]. TLR4 is also different in that it needs the adaptor protein TRAM to link with TRIF, whereas TLR3 only interacts with TRIF to activate the TRIF-dependent signalling pathway [49, 52]. Previous studies in bivalves [64, 65] have shown that both, TLRs and MyD88, are induced by a variety of pathogens. More importantly, *in vitro* studies using TIR-domains of Toll receptor and MyD88 genes from the mussel *Hyriopsis cumingii* showed that the domains were able to induce the expression of antimicrobial peptides, suggesting that a MyD88-dependent signalling pathway is present in molluscs [66, 67]. Considering these results and that our *S*. *glomerata* transcriptomes contain multiple TLR and MyD88 transcripts, it is likely that a MyD88-dependent TLR signalling pathway also exists in *S*. *glomerata*.

#### MyD88-dependent pathway

Once MyD88 has associated with the receptor, IL-1R-associated kinase 4 (IRAK4) is recruited to the receptor complex, with the interaction between IRAK4 and MyD88 leading to the phosphorylation and subsequent activation of IRAK 1 and/or IRAK 2[50, 52]. Phosphorylation of IRAK 1 allows TRAF 6 (tumor necrosis factor [TNF] receptor-associated factor 6) to bind to the complex, after which IRAK 1 –TRAF 6 dissociates from the receptor complex [50, 52, 60]. Under certain circumstances (signalling through TLR1, TLR2 or TLR4), TRAF 6 can move to the mitochondria where it interacts with ECSIT (evolutionarily conserved signalling intermediate in Toll pathways), resulting in the production of mitochondrial reactive oxygen species (ROS) [68]. ORFs coding for ECSIT, TRAF and IRAK were observed in our *S*. *glomerata* transcriptomes (Table 2), indicating that both TLR signalling pathways (MyD88-dependent and signalling through ECSIT) appear to exist in *S*. *glomerata* (Fig 2). This would enable *S*. *glomerata* to trigger the production and release of the important innate immune components ROS and cytokines in response to pathogens. While cytokine release can lead to the activation of other immune cells, ROS can kill pathogens as well as act as a secondary messenger in cellular signalling pathways, such as the MyD88-dependent pathway (involvement in the activation of transcription factors activator protein 1 and nuclear factor-κB) and the RLR signalling pathway [39, 69, 70]. Simulation of both pathways would lead to a strong, protective immune response in *S*. *glomerata*.

In the general signalling pathway, IRAK 1 –TRAF 6 interact with the TAK1 (transforming-growth-factor-β-activated kinase 1)–TAB1 (TAK1-binding protein)–TAB2/TAB3 protein complex, leading to the phosphorylation of TAK1 and TAB2/TAB3. The complex then moves into the cytoplasm of the cell where TAK1 is activated [50, 60, 71]. It has been shown that TAK1 is able to phosphorylate IKKβ (an inhibitor of nuclear factor-κB [IκB]-kinase) and that both, TAB2 and TAB3, appear to be involved in the downstream NF-κB (nuclear factor-κB) activation [50, 71]. Furthermore, it is known that in order to activate NF-κB, IKKα and IKKβ need to be activated by phosphorylation of two serine residues. In addition, along with the catalytically active IKKα and IKKβ, the regulatory subunit NEMO (NF-κB essential modulator) has been shown to be important for NF-κB activation [71]. The active IKK complex (IKKα, IKKβ and NEMO) then phosphorylates IκBα, after which IκBα will eventually be degraded by the proteasome [50, 71, 72]. As IκBα is bound to the inactive NF-κB in the cytosol, phosphorylation of IκBα leads to the release of NF-κB, which then translocates to the nucleus where it actives the transcription of inflammatory cytokine genes [68, 71, 72]. AP-1 (activator protein 1), another transcription factor, can also be activated through the action of TAK1 [49, 52, 68]. AP-1 activation appears to involve a phosphorylation cascade that moves from mitogen-activated protein kinase kinase kinase (MEKK) to MEK to the MAPKs JNK (c-JUN N-terminal kinase) and p38, ultimately leading to the activation of the transcription factor [68]. All the components involved in the MyD88-dependent pathway have been found in our *S*. *glomerata* transcriptomes. Aside from the previously described TLRs, MyD88, IRAK and TRAF transcripts, the *S*. *glomerata* transcriptomes contained ORFs coding for TAK1, TAB1, TAB2, IKK, NF-κB2, NEMO, IκB, AP-1 and a range of MAPKs, MEK, MAP4K and MEKKs (Table 2). This suggests that the MyD88-dependent TLR signalling pathway is conserved in *S*. *glomerata* (Fig 2). Similar results have been seen in high throughput sequencing datasets of mussel (*M*. *edulis*, *Bathymodiolus azoricus*) and oyster (*C*. *virginica*) tissues [25, 26, 54]. Aside from TLRs, MyD88, IκB, TRAF, IRAK and AP-1 were observed in all three studies [25, 26, 54]. In addition, IKK, NF-κB, TAK1 and SARM were found in *C*. *virginica* and *M*. *edulis*, ECSIT in *M*. *edulis* and various mitogen-activated kinases across all three studies [25, 26, 54]. Many of the MyD88-dependent pathway components found in our *S*. *glomerata* study, were also examined in *M*. *galloprovincialis* after a bacterial challenge [65]. The authors analysed the expression levels of IRAK-a and -b, ECSIT, TRAF3, TRAF6, TAK1, IKK-1 and -2, NEMO, IκB-1 and -2, Rel and NF-κB and found no response to bacteria challenge for TAK1. ECSIT and TRAF3 expression was shown to be decreased after Gram-negative challenge, while the rest was found to be up-regulated in response to bacteria [65]. The results of these studies suggest that the TLR signalling pathway in molluscs also functions in innate immunity in response to invading pathogens, using similar pathways to those described in mammals.

#### TRIF-dependent pathway

After interaction of TRIF with its receptor, TRIF associates with TRAF 3 that links TRIF with the IKK-related protein complex IKKε –TBK1 (TRAF-family-member-associated NF-κB activator [TANK]-binding kinase 1). The complex then phosphorylates IRF3 (IFN-regulatory factor 3), which leads to IRF3 dimer formation and its localisation into the nucleus. There, IRF3 triggers the expression of type I IFN genes with the aid of p300 and CBP (cAMP-responsive-element-binding protein [CREB]-binding protein) [50, 60]. Type I IFN in turn appears to be able to increase IFN production through the activation of the JAK-STAT (Janus activated kinase–signal transducer and activator of transcription) signalling pathway and subsequent induction of IRF7 [60].

TRIF has also been shown to activate NF-κB through two pathways, involving TRIF’s N- and C-terminal region. In one pathway, TRAF 6 is recruited to TRIF, which eventually leads to the activation of NF-κB. In the second pathway, NF-κB is activated after TRIF associates with RIP1 (receptor-interacting protein 1) through its C-terminal region [50, 60]. Although TRIF has not been found in our study, it is interesting to note that components of the TRIF-dependent pathways were detected in the *S*. *glomerata* transcriptomes. These components were TBK1, TRAF, IRF, p300/CBP, RIP1, JAK and STAT (Table 2). TBK was also found in *C*. *virginica* and *M*. *edulis*, STAT in *M*. *edulis* and *B*. *azoricus*, and IRF and JAK2 in *M*. *edulis* [25, 26, 54], with RIP-like not induced by bacteria, while the expression level of TRAF3 decreased after Gram-negative challenge in the mussel *M*. *galloprovincialis* [65]. Furthermore, similar to findings in *M*. *edulis* [26], no transcript for interferon was found in our *S*. *glomerata* transcriptomes. However, based on GO annotation results, Bettencourt *et al*. [54] detected interferon in their study on *B*. *azoricus*, indicating that interferon might be expressed in molluscs, even though it was not found in *S*. *glomerata* or *M*. *edulis*. Considering that most components of the TRIF-dependent TLR signalling pathway (excluding TRIF and TRAM) have been found in our study, as well as in other molluscs, it is possible that a TRIF-related pathway exists in molluscs that can be triggered either independently of the adaptor protein TRIF or through the actions of an as yet unknown adaptor protein (Fig 2).

#### Other molecules involved in TLR signalling pathways

While inflammatory responses induced by TLR signalling are an important protective mechanism, an excessive response can be detrimental to the health of the host and therefore needs to be tightly regulated [73, 74]. Aside from cytokines and cytokine receptors, components such as pellino, Toll-interacting protein (TOLLIP), interleukin-1 receptor-associated kinase 1-binding protein 1 (IRAK1BP1), suppressor of cytokine signalling (SOCS), NF-κB inhibitor-like protein 1 (NFKBIL1) and heat shock proteins, all thought to be involved in the regulation of TLR signalling pathways and inflammatory responses, were found in our *S*. *glomerata* transcriptome (Table 2). In mammalian TLR signalling pathways, pellino is thought to act as a type of scaffolding protein by forming a complex with IRAK 1, once the TLRs have been activated through interaction with their ligand. Similar to pellino, TOLLIP appears to carry out its function by interacting with IRAK 1. In non-stimulated cells, TOLLIP inhibits the phosphorylation of IRAK 1, effectively stopping the activation of NF-κB [60]. Different to pellino and TOLLIP, IRAK1BP1 appears to act on NF-κB, resulting in the down-regulation of proinflammatory cytokine transcription [75]. A study in the abalone *Haliotis diversicolor* showed that the strongest expression of IRAK1BP1 was in the haemocytes of this mollusc, with the expression levels of IRAK1BP1 increased after *H*. *diversicolor* was challenged with the bacterium *Vibrio parahaemolyticus* [74]. These results suggest that IRAK1BP1 is involved in the immune response of molluscs. NFKBIL1, another regulatory component is believed to be a member of the IκB family with an NF-κB inhibitory function [76, 77]. SOCS proteins can be induced through the stimulation of TLRs and are able to modulate the sensitivity of immune cells such as macrophages to cytokines and therefore mediate inflammatory responses [73]. Similar to pellino and TOLLIP, heat shock proteins, which can stimulate TLRs by acting as endogenous ligands [42], appear to also be linked to the IKK complex of the TLR signalling pathway. While Hsp 90 seems to have a stabilising function on the kinases, interaction of Hsp 70 with NEMO negatively affects NF-κB signalling [71]. All these components have been found in our *S*. *glomerata* study (Table 2), as well as in the *C*. *gigas* genome (SOCS, NFKBIL1, heat shock proteins) [21], in *C*. *virginica* (pellino) [25], *M*. *edulis* (TOLLIP, SOCS) [26] and *H*. *diversicolor* (IRAK1BP1) [74], indicating that these proteins are also important in molluscs, with a potential function in immunity. Further studies are needed to determine their exact function in the innate immunity of *S*. *glomerata* and other molluscs.

Another molecule of interest that has been connected to several components of the TLR signalling pathway is the adaptor molecule MAVS (mitochondrial antiviral signalling protein), which has also been detected in our *S*. *glomerata* transcriptomes (Table 2). MAVS has been found to be linked to the outer mitochondrial membrane where it functions in antiviral signalling by activating NK-κB, IRF3 and IRF7 in response to viruses. However, the exact mechanism of this activation has not yet been fully elucidated. Current knowledge is that MAVS can interact with cytosolic RIG-I (retinoic acid-inducible gene I, or DDX58) and MDA5 (melanoma differentiation-associated gene 5), which are RIG-I-like receptors that can recognize viruses. MAVS has also been shown to bind TRAFs (e.g. TRAF 6, TRAF 3), as well as interact with TRADD (TNFR1-associated death domain protein) to activate IRF3 and IRF7 through a signalling pathway that involves TRAF3, TANK, IKKε and/or TBK1. NF-κB activation is believed to involve TRADD, FADD (FAS-associated death domain protein) and RIP1, with the kinases IKKα and IKKβ also indicated in MAVS based signalling [78]. While the exact pathway involved in MAVS signalling has not been fully elucidated in mammals, the main components so far associated with MAVS have also been found in our *S*. *glomerata* study. Along with MAVS, ORFs coding for MDA5/DDX58, RIP1 and FADD were detected in our *S*. *glomerata* study (Table 2), suggesting that *S*. *glomerata* could potentially recognise and respond to viruses (Fig 2). While Philipp *et al*. [26] only observed MDA5 and DDX58 but not MAVS in *M*. *edulis*, MAVS was also found in *C*. *gigas*, where its inhibition led to a decrease in TRAF3 expression [79]. The results of this recent study not only showed that MAVS exists in other oyster species aside from *S*. *glomerata*, but also that MAVS expression seems to be linked to some degree with the expression of TRAF3 as has been observed in mammals. With mass mortalities of oysters caused by viral diseases such as *Ostreid herpesvirus-1* (OsHV-1) [80], an effective innate immune response against viruses would be essential for the health and survival of oysters such as *S*. *glomerata*. Considering that mammalian MAVS and RIG-I-like receptors can recognise and respond to viruses, eventually leading to an innate immune response against these pathogens, MAVS might be a promising target for further research in oysters.

### Hypoxia-inducible factors (HIFs)

Oxygen availability is important for vertebrate and invertebrate alike and has roles in many biological functions (e.g. energy production). Oysters such as *S*. *glomerata* are often exposed to hypoxia in their natural habitat (e.g. during low tide or high nutrient load in the water) and need mechanisms that allow it to adapt to a low oxygen environment [81, 82]. HIFs are genes that have been shown to play a role in adaptation to hypoxia in animals [81], with HIF-1α/HIF-2α as well as HIF-1β observed in our *S*. *glomerata* transcriptome (Table 2). While the observation of HIF genes in *S*. *glomerata* would be expected, HIF have recently been linked to the innate immune response in mammals and could potentially have a similar secondary role in *S*. *glomerata* next to their protective function against hypoxia. The heterodimeric HIF functions as a transcription factor, and through the genes whose expression is regulated by HIF, has a role in various cellular pathways such as metabolism, cell differentiation and apoptosis [83]. HIF is comprised of two subunits, HIF-α and HIF-1β, with HIF-α encompassing the following 3 genes: HIF-1α, HIF-2α and HIF-3α [84]. In a normoxic environment and without an activating stimulus (e.g. insulin-like growth factor, interleukin-1β or LPS), HIF-α is quickly degraded by prolyl hydroxylases (PHDs) or otherwise regulated by hypoxia-inducible factor 1-alpha inhibitor (HIF1AN) [83, 85]. However, bacterial LPS for instance, has been shown to be able to increase HIF-1α mRNA expression not only in hypoxic but also in normoxic conditions [86, 87]. This LPS induction of HIF-1α expression appears to involve TLR4 and p44/42 MAPK and NF-κB signalling pathways [83, 86, 87]. Under hypoxic conditions, for example during inflammation/infection where the metabolism of pathogens and host inflammatory cells and decreased perfusion creates a localised low oxygen environment, PHDs and HIF1AN are inhibited and HIF-α associates with p300-CBP (CREB-binding protein). HIF-α–p300-CBP then moves to the nucleus where the HIF heterodimer forms and the HIF complex binds to the hypoxic-response elements (HREs), resulting in the regulation of target gene expression. In addition, HIF is thought to boost neutrophil and macrophage migration to areas of infection, PAMPs detection, phagocytosis, bactericidal activity (e.g. antimicrobial peptides, tumour necrosis factor and nitric oxide) and the survival of neutrophils, macrophages and monocytes [83–85]. HIF-1 has also been indicated in the transcriptional regulation of TLR4 expression in macrophages exposed to hypoxia and in the direct regulation of TLR2 and TLR6 expression [88, 89]. The *S*. *glomerata* reference transcriptomes of this study contain ORFs for all components of the HIF pathway, from HIF-1α/ HIF-2α, HIF-1β, HIF1AN to PHD and p300/CBP (Table 2) based on sequence homology as well as InterProScan domain and family matches. Some of these genes have also been found in other molluscs, such as HIF-α and PHD that have been cloned from *C*. *virginica* and *C*. *gigas* [81, 82] and HIF1AN that was found in the *C*. *gigas* genome [21]. However, to the best of our knowledge, HIF-1β has not been previously found in oysters and is therefore reported here for the first time in the oyster *S*. *glomerata*. While most research into HIF’s roles in immunity appears to have been carried out in vertebrates, one study in *C*. *gigas* has observed a link between HIF-α expression and respiratory burst activity in haemocytes, indicating that HIF-α has a role in reactive oxygen species (ROS) production in this oyster species [90]. Although HIF’s main role might be in allowing *S*. *glomerata* to adapt to short-term hypoxia, the study in *C*. *gigas* suggests that HIF could also have a role in immunity, similar to mammalian HIF (Fig 2). Furthermore, with ORFs for all components of the HIF pathway found in the *S*. *glomerata* transcriptomes, as well as ORFs for the TLR signalling pathway, a dual role of HIF in *S*. *glomerata* could be possible (Fig 2). Further research is necessary to determine HIF’s exact role(s) in *S*. *glomerata* and other oyster species. Sequence information gained in our transcriptome study could be beneficial for such future research endeavours.

### Phagocytosis and antioxidant defence

Phagocytes, such as macrophages are an important part of the innate immune defence in response to microorganisms and phagocytic activity of immune cells has been shown not only in vertebrates, but also in invertebrates such as *M*. *galloprovincialis* and *S*. *glomerata* [11, 91, 92]. In these and other bivalves, phagocytosis is the most important cellular defence mechanism, with a large number of immune cells (2–4 x 10^6^ cells/ml) contained within the haemolymph [46, 92]. The basic principle behind phagocytosis is the recognition of microorganisms or cells, their internalisation and destruction [91]. Recognition of particles occurs through a variety of receptors, for example, TLRs and other PRRs (e.g. mannose receptor), opsonic receptors (e.g. complement receptors) or apoptotic corpse receptors (e.g. stabilin-2) [91, 93]. Some of these receptors, such as TLRs, macrophage mannose receptors, SRs and the apoptotic corpse receptor stabilin-2 have also been found in our *S*. *glomerata* transcriptomes (Table 2). Receptors that bind to immunoglobulin G (IgG) (e.g. FcγRI) or IgAs (FcαRI), however, have not been found in *S*. *glomerata* and also do not appear to have been observed in the *C*. *gigas* genome [21]. As oysters do not possess adaptive immunity, it is likely that these receptors are not used in oysters. The receptors found in *S*. *glomerata* (e.g. TLR, SRs) could potentially fulfil the important innate immune function of foreign particle recognition for phagocytic clearance, therefore ensuring that the host is protected from potentially harmful microorganisms or accumulating apoptotic bodies (Fig 2). Furthermore, as described previously, a ficolin-like recombinant protein from *C*. *hongkongensis* was shown to increase the phagocytic activity of *C*. *hongkongensis* haemocytes by acting as an opsonin [55]. Having these multiple receptors that directly or indirectly (e.g. through opsonins) result in the successful phagocytic clearance of cells or invading microbes would be beneficial for *S*. *glomerata* as these oysters are continuously exposed to bacteria in their natural environment and would therefore need a strong protective immune mechanism such as phagocytosis to maintain their health.

Depending on the receptor(s) that recognise the particles, specific signalling pathways are triggered that eventually lead to the engulfment of the particles and their internalisation into the phagosome [93]. The phagosome then undergoes maturation in which it becomes increasingly more acidic before it fuses with lysosomes to form a phagolysosome [93]. Along with the acidic (pH 4.5–5.0) environment, phagolysosomes contain active cathepsins, ROS (e.g. superoxide anions, hydrogen peroxide and hydroxyl radicals), reactive nitrogen intermediates (RNI) and antimicrobial proteins and peptides (e.g. lysozyme, defensins) that allow phagocytes to destroy the engulfed targets [69, 93]. Enzymes that are involved in the production of ROS and RNI as well as ORFs coding for cathepsins, antimicrobial proteins and peptides have been found in the *S*. *glomerata* transcriptome of this study (Table 2) and are discussed in detail in the following sections.

#### ROS and RNI

In response to proinflammatory cytokines or PAMPs, phagocytes express nitric oxide synthase (NOS) which catalyses the reaction that produces nitrous oxide. Nitrous oxide then reacts with ROS to create a range of potent RNI (e.g. peroxynitrite) that are effective against lipids, proteins and nucleic acids [70, 93]. NOS activity has been detected in a variety of molluscs, for example, in the haemocytes of the freshwater snail *Viviparus ater* and the mussel *M*. *galloprovincialis* and NOS sequences have been found not only in our study (Table 2) but also in *A*. *californica* and other marine invertebrates [94–96]. With NOS activity observed in other molluscs, it is likely that the NOS found in this *S*. *glomerata* study also functions in the production of nitrous oxide.

ROS, which can interact with nitrous oxide to form toxic RNI, are produced as a by-product of biological reactions as well as specifically formed by phagocyte NADPH oxidase (NOX). To date, seven NOX isoforms have been discovered, NOX 1–5, dual oxidase 1 (DUOX 1) and DUOX 2, with all members of the family functioning as electron transporter, reducing oxygen to superoxide in the process [97]. Similar to mammalian studies, multiple transcripts coding for NOX and DUOX have been found in the *S*. *glomerata* of our study (Table 2). In addition, DUOX has been found in the *C*. *gigas* genome [21] and also in the scallop *Mizuhopecten yessoensis*, where DUOX expression was found to be induced in the gill after exposure to copper [98]. While NOX 2 has also been shown to be expressed in non-phagocytic cells, its function appears to be the production of ROS in phagocytes [70, 93, 97]. Proinflammatory stimuli trigger the formation of the NOX 2 (also known as gp91^phox^) complex in phagocytes that forms between NOX 2 and p22^phox^, a transmembrane protein. Once the two proteins have associated with each other to form flavocytochrome b_558_ the complex is activated by cytosolic components (p47^phox^, p67^phox^ and p40^phox^) that translocate once p47^phox^ has been phosphorylated, allowing p47^phox^ to interact with p22^phox^ [93, 97]. Another important molecule NOX 2 interacts with during complex assembly and activation is the small GTPase Rac [93, 97], which has also been detected in the transcriptomes of this study (Table 2). As one transcript coding for gp91^phox^ (NOX 2) has also been found in our study (Table 2), it is possible, that NOX 2 and Rac could interact in a similar way with each other in *S*. *glomerata* as it does in mammals, potentially resulting in the production of ROS.

Aside from NOX 2, mitochondria can also produce ROS that aid macrophages in their bactericidal activity. Mitochondrial ROS is generated through TLR signalling (TLR1, TLR2 and TLR4) during which TRAF6 translocates to the mitochondria where it interacts with ECSIT (evolutionarily conserved signalling intermediate in Toll pathways), leading to a rise in mitochondrial ROS production [78]. One ORF coding for a 451 amino acid long ECSIT has been detected in this study (Table 2), as well as in *C*. *gigas*. In their study, Zhang *et al*. [99] showed ECSIT expression levels were significantly up-regulated in the haemolymph of *C*. *gigas* challenged with the bacterium *V*. *anguillarum* when compared to control oysters, indicating that ECSIT plays a role in innate immune defence of oysters. Additionally, the authors observed ECSIT gene expression in the haemolymph, gill, digestive gland, muscle, mantle and gonad of *C*. *gigas* [99]. This is in accord with our data, where the ECSIT transcript was detected in the haemolymph, gill, mantle, adductor muscle, gonad and digestive system of *S*. *glomerata*. Furthermore, comparison of the *C*. *gigas* ECSIT with the *S*. *glomerata* ECSIT, using Clustal Omega showed that the 452 amino acid long *S*. *glomerata* ECSIT shared a 72.3% sequence identity with the *C*. *gigas* ECSIT (S4 Fig) and a 38.2% sequence identity with a *M*. *galloprovincialis* ECSIT (AHI17287). Based on the results of the study by Zhang [99], as well as the sequence identity of the *C*. *gigas* and *S*. *glomerata* ECSIT, it is likely that *S*. *glomerata* ECSIT also functions in innate immune defence (Fig 2).

In addition to Rac, ECSIT, NOX, DUOX and gp91^phox^, p22^phox^ and p67^phox^ have also been found in our study (Table 2). Interestingly, based on amino acid (aa) length, *S*. *glomerata* NOX and NOX-related genes appear to be very similar to the mammalian orthologous genes. For instance, mammalian NOX are 564–747 aa long [97] and *S*. *glomerata* NOX between 568 and 851 aa. Similarly, *S*. *glomerata* DUOX (396 to 1,627 aa, with the majority between 1,392–1,627 aa), gp91^phox^ (568 aa), p22^phox^ (172 aa) and p67^phox^ (185 aa—partial sequence) transcripts are comparable to DUOX (1,548–1,551 aa), gp91^phox^ (570 aa), p22^phox^ (195 aa) and p67^phox^ (526 aa) gene length in mammals [97]. Research into non-mammalian species indicates that ROS production by NADPH oxidase might not be limited to mammals. For instance, Adema *et al*. [100] used haemocytes of the pond snail *Lymnaea stagnalis* to test inhibitors of NADPH-oxidase and compounds that inhibit NOX complex assembly in mammalian cells and showed that phagocytosis was inhibited by these compounds in the pond snail. Another study assessed the effect of *V*. *splendidus* challenge of *C*. *gigas* haemocytes on the expression of genes involved in phagocytosis and observed a significant increase of NADPH oxidase after a secondary challenge with the bacterium [101]. While these studies suggest that NADPH oxidase also produces ROS in non-mammalian cells, we only found an ORF for one of the three cytosolic components (p67^phox^) of the NOX2-complex in our transcriptome, but no ORFs for p47^phox^ or p40^phox^. In addition, no apparent hits for p40^phox^ (alternative name: SH3 and PX domain-containing protein 4) or p47^phox^ (alternative name: SH3 and PX domain-containing protein 1A) could be found in the *C*. *gigas* genome [21]. This suggests that these two cytosolic components might not be needed for NOX2-complex formation in oysters. Alternatively, other molecules not yet found might carry out the roles of p40^phox^ and p47^phox^ in oysters, or the sequences have diverged in molluscs and therefore could not be detected in our *S*. *glomerata* study. Further research is needed to elucidate the exact process by which the NOX2-complex forms and functions in oysters. In addition, while one p22^phox^ was observed in the *C*. *gigas* genome [21], p67^phox^ and gp91^phox^ do not appear to have been found in any other oyster yet and have been reported for the first time in our *S*. *glomerata* study.

Considering the environment *S*. *glomerata* inhabit, immune components with strong bactericidal activity (e.g. ROS) are essential to the health and survival of these bivalves. In this study we have found genes necessary to produce both nitrous oxide and ROS. Furthermore, fundamental components of two ROS production pathways were found in *S*. *glomerata*: NOX 2 and ECSIT. Possessing two pathways that potentially lead to the synthesis of ROS could allow *S*. *glomerata* oysters to produce sufficient amounts of ROS, nitrous oxide and RNIs to successfully protect themselves from pathogens (Fig 2). Furthermore, ROS production by the mitochondria through TLR signalling, whose individual components have also been found in this study, would allow *S*. *glomerata* to directly respond to endogenous (stress related) or exogenous (bacterial challenge) ligands (Fig 2).

#### Antioxidants

While RNI and ROS are an important part of the innate immune defence mechanism, ROS have also been shown to play roles in, for instance, cellular signalling, apoptosis, cell growth and differentiation. However, while ROS can be beneficial, a fine balance has to be maintained of the level of ROS produced and removed, as any disruption in the balance can lead to oxidative stress which may result in damage to a variety of macromolecules like DNA, RNA and proteins [39, 40, 69]. This balance is maintained by non-enzymatic (e.g. minerals and carotenoids) and enzymatic antioxidants such as superoxide dismutase (SOD), glutathione peroxidase (GPX) and catalase (CAT), as well as redox proteins like thioredoxin, glutaredoxin and peroxiredoxin, which allow the host organism to protect itself from the harmful effects of ROS [39, 40, 102]. In this *S*. *glomerata* transcriptome study, ORFs encoding the proteins SOD, GPX and CAT were found (Table 2). These three enzymatic antioxidants protect organisms from oxidative stress, caused by ROS such as superoxide anion and hydrogen peroxide (Fig 2). SOD, which exists in three forms in humans (cytosolic copper/zinc-SOD, mitochondrial manganese-SOD and extracellular SOD), reacts with superoxide anions, generating hydrogen peroxide in the process. Hydrogen peroxide in turn is catalysed by CAT and GPX, resulting in the formation of water and molecular oxygen. Whereas both CAT and GPX can remove hydrogen peroxide, only GPX needs glutathione (GSH) to catalyse the reaction [39, 40]. GSH is synthesised in the cytosol from glutamate, cysteine and glycine through the sequential catalytic action of the two enzymes γ-glutamylcysteine synthetase (GCS) that consists of a catalytic (also called glutamate—cysteine ligase catalytic subunit) and a regulatory polypeptide (also called glutamate—cysteine ligase regulatory subunit) and glutathione synthetase (GS) [103, 104]. Once GSH has been oxidised to glutathione disulfide (GSSG) during the reduction of hydrogen peroxide, it can be reduced back to GSH by the action of glutathione reductase [39]. These enzymes (GS, both GCS subunits and glutathione reductase) involved in the synthesis and reduction of GSH have also been found in the transcriptomes of this study (Table 2), indicating that GSH synthesis and GSSG reduction are conserved and also provide the necessary GSH for the removal of hydrogen peroxide in *S*. *glomerata*. In addition, we have also found thioredoxin, thioredoxin reductase, peroxiredoxin, glutaredoxin, glutathione S-transferase (GST) and two methionine sulfoxide reductases (MSRs) in the *S*. *glomerata* transcriptomes (Table 2). The thioredoxin system is another major antioxidant system that functions, among others, in oxidative stress defence by transferring electrons to, for instance peroxiredoxin, that is able to react with and remove H_2_O_2_, ROOH and ONOO^-^ [39, 105]. Reaction efficiency of peroxiredoxin in terms of H_2_O_2_ removal appears to be on par with GPX and CAT. Once peroxiredoxin has disposed of H_2_O_2_, it is reduced to its active state by the thioredoxin antioxidant system. Similar to peroxiredoxin, MSRs also receive electrons from the thioredoxin system; however, MSRs function only indirectly as ROS scavengers. Oxidative stress can oxidise methionine to methionine sulfoxide, impacting on the protein’s function. MSRs are able to transform methionine sulfoxide back into methionine, restoring protein function [105]. More importantly, one MSR was found to be up-regulated in the clam *Ruditapes decussates* in response to the parasite *Perkinsus oleni*, indicating that MSR is involved in the immune response of this mollusc [106]. Oxidised thioredoxin itself can be reduced by thioredoxin reductase, as well as by GSH and glutaredoxin. In turn, the thioredoxin system appears to be able to reduce oxidised GSH, interlinking the GSH and thioredoxin antioxidant systems [105]. Another more indirectly acting antioxidant family are GSTs that protect against molecules, such as epoxides and hydroperoxides produced by oxidative stress [103].

Of the antioxidants and antioxidant related genes found in the *S*. *glomerata* transcriptomes of this study, peroxiredoxin, GPX and GST have also been detected in Akoya pearl oysters (*Pinctada fucata*), when the oysters were exposed to stressors such as mechanical agitation and air [107]. Green *et al*. [108] also found peroxiredoxin 6 and SOD differentially expressed in *S*. *glomerata* selected for disease resistance when compared to wild oysters. In addition, CAT and GPX have been cloned in *C*. *gigas* and their expression and activity along with SOD measured in *C*. *gigas*, *Saccostrea cucullata*, and *Bathymodiolus azoricus* [109–111]. Furthermore, GSH levels have been measured in *S*. *glomerata* and *C*. *gigas* [112, 113]. Taken together, these results suggest that the main antioxidants CAT, SOD and GPX might function similarly in vertebrates and invertebrates such as oysters. In addition to the main antioxidants, the activity of glutathione reductase, thioredoxin reductase and GST was measured in the gills of *C*. *gigas* [113] and an EST for thioredoxin found in *C*. *virginica* [114]. Expressed sequence tags were found for GST, thioredoxin, thioredoxin reductase, GPX and SOD in *M*. *galloprovincialis* exposed to stressors such as pollutants, bacteria and temperature [115]. Furthermore, sequences for thioredoxin reductase, glutaredoxin, GS, both glutamate—cysteine ligase subunits, glutathione reductase and both MSRs were also found in the *C*. *gigas* genome [21]. These and our *S*. *glomerata* results show that antioxidants and antioxidant related genes are conserved across a wide range of molluscs, indicating that antioxidant defence is an important mechanism for *S*. *glomerata* and other molluscs. Moreover, taking into account the complex set of antioxidant and antioxidant related genes found in our *S*. *glomerata* transcriptomes, antioxidant defence appears to be well developed in this oyster species. This will allow *S*. *glomerata* oysters to guard themselves against the detrimental effects of ROS and RNIs, while benefiting from their protective functions. Additionally, as *S*. *glomerata* appear to not only have the GSH but also the thioredoxin system and GSTs to defend themselves from oxidative stress, they might still be sufficiently protected should a specific pathway or component be inhibited (Fig 2).

#### Proteases and antimicrobial proteins and peptides

Among the defensive arsenal of host cells are cysteine (cathepsins B, C, F, H, K, L, O, S, V, X and W), aspartic (cathepsins D and E) and serine proteases (cathepsins A and G), whose gene expression is controlled by a range of inflammatory stimuli [41, 116, 117]. The main function of cathepsins in the lysosomal system is the degradation of proteins; however, their proteolytic activity depends on the pH of their environment [116, 117]. While most studies into cathepsins have been carried out in mammals, 59 transcripts coding for cathepsins have also been found in this *S*. *glomerata* transcriptome study (Table 2), as well as in the haemocytes of *C*. *virginica* and the mussel *Cristaria plicata* [114, 118]. Furthermore, cathepsin B and C expression was shown to be induced in the clam *Sinonovacula constricta* by the bacterium *V*. *anguillarum* [119, 120]. These results suggest that cathepsins could also play an important role in the innate immune defence of oysters and other molluscs. Moreover, the range of cathepsins found in our *S*. *glomerata* transcriptomes indicates that these proteases are an important component of the *S*. *glomerata* innate immunity (Fig 2).

In addition to cathepsins, the *S*. *glomerata* transcriptomes contained transcripts coding for lysozyme, big defensin, hydramacin and bactericidal permeability increasing protein (BPI) (Table 2). Lysozymes are hydrolytic enzymes that act on bacterial peptidoglycan to destroy bacterial pathogens, with cDNA sequences for this enzyme also isolated from a variety of other molluscs, such as *Bathymodiolus thermophilus*, *Calyptogena* sp. 1, *M*. *galloprovincialis*, *Mactra veneriformis* and others [121, 122]. Gene expression levels of an i-type lysozyme increased in the mussel *C*. *plicata* after exposure with *Aeromonas hydrophila*, and the recombinant lysozyme produced in the same study demonstrated bacteriolytic activity against a range of bacteria (e.g. *A*. *hydrophilia*, *Bacillus subtilis*, *Staphylococcus aureus* and *Escherichia coli*) [123]. Moreover, Zhao *et al*. [124] cloned a lysozyme from the scallop *Chlamys farreri* with sequence similarity to g-type lysozymes. The authors also assessed the lytic activity of the recombinant lysozyme protein, showing that the protein was bactericidal against the Gram-positive bacteria *Micrococcus lysodikicus* and *Micrococcus luteus*, with only weak lytic activity against Gram-negative bacteria (e.g. *V*. *parahaemolyticus* and *V*. *anguillarum*) and no activity against *E*. *coli* and *S*. *aureus* [124]. These studies showed that not only vertebrate, but also invertebrate lysozymes have a bactericidal ability against a wide range of bacterial targets. Considering that invertebrates do not have adaptive immunity, expressing lysozymes with lytic activity towards a variety of bacterial pathogens could allow *S*. *glomerata* oysters to protect themselves from the bacteria they encounter in their natural environment.

Aside from lysozymes, the invertebrate innate immune defence system also depends on antimicrobial peptides (AMPs) like defensins. These cationic antimicrobial peptides carry out their defence function by forming pores in the bacterial membrane, which ultimately leads to an osmotic imbalance in the bacterial cell [93, 125]. Research into big defensins in the haemolymph of horseshoe crabs indicated that this invertebrate AMP is active against fungi as well as Gram-positive and Gram-negative bacteria [126]. A similar pattern was observed in the scallop *Argopecten irradians*, where big defensin was shown to be active against Gram-positive bacteria, weakly against Gram-negative bacteria (no effect on *E*. *coli*), and a potential fungicidal activity against the yeast *Pichia pastoris* [127]. Furthermore, big defensin expression levels increased in the haemolymph of the clam *Venerupis philippinarum* after *V*. *anguillarum* challenge. In addition, big defensin also showed an inhibitory activity against other Gram-negative and Gram-positive bacteria [128]. Interestingly, one study assessed the expression levels of three big defensins in the haemolymph of *C*. *gigas* and found two of them up-regulated in response to bacterial challenge. It was hypothesised by the authors that the third big defensin might be constitutively expressed in the haemocytes of this oyster [126]. Together these results suggest that big defensins in invertebrates are active against a wide range of targets and that not all big defensins might be regulated in the same manner. When the *S*. *glomerata* big defensins of this transcriptome study were compared with each other, two of the five big defensin ORFs (m.22175 and m.22176) found in this transcriptome showed 87% sequence identity with each other, but only between 33 to 48% with the other three ORFs (m.6735, m.28084 and m.24530) (S5 Fig). These results show that *S*. *glomerata* big defensin protein sequences display a fairly high variability. Interestingly, the five *S*. *glomerata* big defensins also appeared to be expressed in different tissues. While transcripts of three of the big defensins (m.22175, m.22176 and m.24530) were expressed in all six tissues, m.6735 was only expressed in the digestive tissue and transcripts of m.28084 were detected in all tissues but the gill and adductor muscle. It is unclear why some *S*. *glomerata* big defensins appear to be only expressed in some but not all of the tested tissues. Nevertheless, it was interesting to note that only the *S*. *glomerata* digestive tissue had all five big defensins expressed. Considering that the particles that are filtered from the water column by *S*. *glomerata* also contain bacteria, it might be beneficial for this oyster to have an as broad as possible range of big defensins expressed in the digestive system to protect itself from ingested bacteria. Alignment of the five *S*. *glomerata* big defensins to the putative protein sequences of the big defensins of the scallop *A*. *irradians*, clam *V*. *philippinarum* and *C*. *gigas* studies above showed that *S*. *glomerata* big defensins mapped closer to the big defensins of *C*. *gigas* (sequence identities of 27–30% with m.6735, 46–54% with the other four) than to the big defensins of *V*. *philippinarum* and *A*. *irradians* (sequence identities between 22% (m.6735) and 18–35%) (S5 Fig). This was expected to be seen as *S*. *glomerata* are more closely related to *C*. *gigas* then to either clam or scallop.

Bactericidal permeability increasing protein (BPI), another AMP found in our study (Table 2), has been shown to counteract the effects of LPS (e.g. TNF-α production), has anti-inflammatory properties and a role in opsonisation that leads to an increase in phagocytosis. In addition, BPI expression can be triggered by LPS (TLR—MyD88-independent pathway) and carries out its bactericidal activity by causing damage to the bacterial membrane [129, 130]. This antimicrobial peptide has been identified in *C*. *gigas* haemocytes, where the recombinant protein was able to bind LPS, damage bacterial membranes and had antibacterial activity against the strain *E*. *coli* SBS363. BPI expression levels were also observed to increase in response to bacterial challenge [130], indicating that *C*. *gigas* BPI had similar functionality to vertebrate BPI. While the majority (18) of the *S*. *glomerata* BPIs appear to be expressed in all six tissues studied, six of the BPIs were expressed in all tissues but the adductor muscle, and one each were expressed in a) haemolymph, gill and mantle, b) haemolymph and mantle and c) all but adductor muscle and digestive tissue. Hydramacin, an AMP detected for the first time in the clam *V*. *philippinarum* [131] was also expressed in all *S*. *glomerata* tissues of this study. This coincides with the tissue expression pattern of hydramacin in *V*. *philippinarum*, where it was found in the adductor muscle, mantle, gill, haemocytes, shiphon and foot of the clam [131]. Similar to other AMPs, hydramacin transcript expression was shown to increase after clams were challenged with the bacterium *Vibrio tapetis* [131]. Alignment of the *S*. *glomerata* and *V*. *philippinarum* hydramacin showed a sequence identity of 37% (S6 Fig) indicating that the two putative protein sequences are not closely related.

Considering that *S*. *glomerata* oysters are directly exposed to a variety of microorganisms in their natural environment, phagocytosis with its various protective components (e.g. AMPs, hydrolytic enzymes) is an important innate immune defence mechanism that allows the animals to combat the harmful effects of invading pathogens (Fig 2). Furthermore, expressing more than one type of AMP in different tissues, as has been seen in this study, would allow *S*. *glomerata* oysters to protect themselves from a diversity of bacterial pathogens. Future research into expression levels of different AMPs and different types of the same AMP in *S*. *glomerata* in response to stress or bacterial challenge would give us a more detailed view on the protective function of AMPs in this animal, with potential implications for other molluscs.

### Apoptosis

Apoptosis is an evolutionary conserved mechanism with essential functions in homeostasis and innate immunity that removes damaged cells without triggering inflammation [132, 133]. This was shown in a study in *C*. *virginica*, where a basic level of apoptosis of up to 25% was observed in circulating haemocytes. Furthermore, infection of the oyster with the parasite *Perkinsus marinus* resulted in increased haemocyte apoptosis levels, indicating that apoptosis is an important immune response in oysters such as *C*. *virginica* that allows the immune system to remove infected haemocytes [134]. Current knowledge suggests that vertebrate and invertebrate apoptosis share many components of apoptotic cascades, with detailed descriptions of the apoptotic cascades in vertebrates and to some degree in molluscs available [132, 135, 136]. In short, the apoptotic signalling cascades are generally split into two interconnected pathways, an intrinsic and an extrinsic cell death pathway [135, 136], with environmental stressors (e.g. pollution, salinity fluctuations) believed to stimulate the intrinsic pathway in molluscs [132]. Induction of the intrinsic pathway involves pro-apoptotic proteins (e.g. Bax and interferon alpha-inducible protein 27 [IFI27]) and results in the release of proteins (cytochrome c, Smac/DIABLO [second mitochondrial activator of caspases/direct IAP-binding protein of low isoelectric point] and AIF [apoptosis-inducing factor]) from the mitochondria into the cytosol [135, 137]. These mitochondrial factors interact with other components of the signalling cascade (e.g. apoptotic protease-activating factor-1 [Apaf-1], caspases) to promote apoptosis. Baculoviral IAP repeat-containing proteins (IAPs) inhibit caspase activity, with the inhibitory action of IAPs removed through binding with Smac/DIABLO. AIF in comparison causes apoptosis in a caspase-independent manner [135]. Our *S*. *glomerata* transcriptomes contained multiple transcripts of caspases, IAPs, DIABLO and one transcript for AIF, along with transcripts for the two pro-apoptotic proteins, Bax and IFI27 (Table 2). Similar to our study, caspases and AIFs were found in *P*. *maximus* and *M*. *edulis* [26, 53], caspases, IAPs and Bax in *C*. *virginica* [25], IFI27 in *C*. *gigas* [21] and DIABLO in *A*. *californica* [GenBank:XP_005110230] (http://www.ncbi.nlm.nih.gov). While these results show that many of the components of the intrinsic apoptosis pathway are also found in molluscs, we did not find any transcripts for Apaf-1 in *S*. *glomerata*. Moreover, to the best of our knowledge, no Apaf-1 transcripts have been observed yet in any other mollusc. This suggests that while the majority of the intrinsic pathway appears to be conserved in *S*. *glomerata*, this pathway either acts independent of the action of Apaf-1 or contains an as of yet unknown component with similar functions to Apaf-1 in mammals.

Apart from the intrinsic apoptosis pathway, the extrinsic apoptosis pathway also seems to exist in molluscs [136]. Ligand binding (e.g. TNF) to a death receptor of the TNF receptor superfamily induces apoptosis through the extrinsic pathway that involves the recruitment of FAS-associated death domain protein (FADD) and the action of several caspases [135, 136]. These components of the extrinsic pathway have also been detected in our *S*. *glomerata* transcriptome, with ORFs potentially coding for FADD, TNF receptor superfamily members and TNF superfamily members (Table 2). FADD, TNF and TNF receptor superfamily members have also been found in *C*. *virginica* [25], indicating that these proteins are important in oysters. Overall, having found components of both, the intrinsic and extrinsic cell death signalling pathways, in our *S*. *glomerata* transcriptomes, suggests that apoptosis is an integral part of the *S*. *glomerata* immune response to maintain its health (Fig 2).

## Conclusions

In this study, we have exposed the economically and ecologically important oyster species *S*. *glomerata* to a range of environmental stressors to obtain sequencing data for six different tissues (haemolymph, gill, mantle, adductor muscle, gonad and digestive). Many of the transcripts expressed in *S*. *glomerata* were found across all six tissues, with a smaller number of transcripts specifically expressed in the haemolymph of the oyster, suggesting a function in the innate immune system of *S*. *glomerata*. Some of the transcripts exclusive to the haemolymph were involved in phagocytosis (e.g. cathepsins), antioxidant defence (e.g. SOD, peroxiredoxin) and potentially TLR signalling (e.g. heat shock proteins). Closer examination of the *S*. *glomerata* transcriptomes revealed a wide range of transcripts potentially involved in the innate immune defence against invading pathogens. A schematic visualising the innate immune arsenal stimulated in *S*. *glomerata* in response to stress is shown in Fig 2. Pathways such as TLR signalling or apoptosis appear to be largely conserved in *S*. *glomerata*, indicating that these pathways are also important in invertebrates such as oysters. In addition, HIF genes, generally associated with hypoxia, but recently also linked to immunity in mammals, have been found in *S*. *glomerata*, with HIF-1β reported for the first time in oysters. Components of other equally important immune functions such as phagocytosis and antioxidant defence were also observed in *S*. *glomerata*, with to the best of our knowledge p67^phox^ and gp91^phox^ reported for the first time in oysters. Overall, this study examined for the first time the complex machinery of the *S*. *glomerata* innate immune system and will be a valuable source for further research into the innate immune system of *S*. *glomerata* and other oyster species.

## Methods

### Illumina sequencing

Wild, adult *S*. *glomerata* oysters were collected from Cromarty Bay (NSW, Australia) and exposed to a variety of environmental stressors in a controlled setting (S1 File). Different levels of a) CO_2_ and temperature (n = 90 oysters), b) salinity and temperature (n = 174 oysters), c) copper (n = 24 oysters) and d) pyrene and fluoranthene (n = 63 oysters) were chosen as experimental stressors. In addition, wild and selectively bred (for fast growth and disease resistance) adult oysters were exposed to different levels of CO_2_, spawned and their offspring also exposed to different levels of CO_2_ as larvae and adult. This was done for three generations of oysters. Six tissues (haemolymph, gill, mantle, adductor muscle, digestive system and gonad) were extracted from each of the third generation adult offspring (n = 108 oysters) from this experiment, from the wild oysters of the above four experiments, and from non-stressed control oysters at multiple time-points throughout the experiments. Small amounts of tissue were pooled per tissue type from all collected samples (n = 459 oysters total) and total RNA extracted, using TRIsure^™^ (Bioline, Australia) according to the manufacturer’s protocol. Quantity and quality of the total RNA was tested with the NanoDrop2000 spectrophotometer (Thermo Fisher Scientific, USA) and gel electrophoresis before samples were freeze dried and sent to BGI (Hong Kong) for sequencing. On arrival, BGI checked the quality of the total RNA samples with an Agilent Bioanalyzer^®^, carried out a DNase I digestion for each of the samples, then prepared strand-specific and normalised, as well as non-normalised and non-strand specific libraries for paired-end 101 bp sequencing for each of the six pooled tissue samples, using Illumina HiSeq-2000. Normalisation of the strand-specific libraries was chosen to increase the possibility of detecting low expressed genes. This, in addition to sequencing non-normalised and non-strand specific libraries, coupled with using tissue from non-stressed and stressed oysters, should result in a most comprehensive transcriptome. Raw sequencing reads were deposited in the NCBI sequence read archive under the BioProject number PRJNA272756.

### Quality control and *de novo* assembly

Quality of the sequencing data of the six tissues was assessed with FastQC (http://www.bioinformatics.babraham.ac.uk/projects/fastqc/) pre- and post-trimming, after which adapter sequences were removed and the sequences trimmed with Trimmomatic [138], using the following parameters: TRAILING:3, MINLEN:36 and HEADCROP:1. Furthermore, the sequences were filtered for artefacts such as *E*. *coli*, plasmid or human sequences, as well as ribosomal sequences. Post-processed reads of the six tissues were combined into two reference data sets and both sets assembled with Trinity [31]. The two data sets were comprised of: a) strand-specific and normalised reads only and b) non-normalised and non-strand specific reads and reverse complement of strand-specific and normalised reads, with the resulting two reference assemblies considered as strand-specific transcriptome and combined transcriptome, respectively throughout the rest of the text. Redundancy was removed from both data sets with cd-hit-est [139], using a 99% cut-off, and potential coding regions determined with TransDecoder (http://transdecoder.sourceforge.net/) and used for further analysis. Completeness of both non-redundant assemblies was assessed with CEGMA (Core Eukaryotic Genes Mapping Approach) [32].

### Functional annotation and tissue distribution

Blastp similarity searches were carried out on the open reading frames (ORFs) of both transcriptomes against the NCBI non-redundant (nr) database (downloaded 01 October 2013) with an e-value cut-off of 1e^-5^ and a hit number threshold of 25. Blast2GO [140] with default parameters (hit adjusted to 25) was used for mapping and annotation of the ORFs, with InterProScan results merged with the already existing annotations. Furthermore, Kyoto Encyclopedia of Genes and Genomes (KEGG) pathway annotations were also obtained via Blast2GO. To obtain information on the transcript expression pattern across different tissues, reads of the six individual tissues were mapped back to the non-redundant strand-specific and combined reference transcriptomes, using the CLC Genomics Workbench version 7.5 (CLC Bio, USA) with default parameters except for length and similarity fraction that were set to 1.0 and 0.9, respectively. General transcript patterns across the six tissues were determined, with the tissue distribution of specific transcripts determined based on the CLC mapping information and the results presented using Microsoft Excel graphs.

### Sequence comparison to *Crassostrea gigas*

Non-normalised and non-strand specific and normalised and strand-specific reads were mapped to the *C*. *gigas* genome [21] with Bowtie 2 [141]. Furthermore, both non-redundant reference transcriptomes were mapped to the *C*. *gigas* genome with CLC Genomics Workbench version 7.5 to determine similarity at the nucleotide level. *S*. *glomerata* transcripts had to match over their entire length with a similarity of at least 60% to the *C*. *gigas* genome to be considered a valid alignment.

### Immune and immune-related genes

In the context of this study, only *S*. *glomerata* ORFs with the following characteristics were considered potential immune genes: 1) sequence homology to known immune and immune-related genes, and 2) annotated with an immune or immune-related GO term (e.g. GO:0006955, GO:0016265 and GO:0006950) and/or containing typical domain organisations as determined with InterProScan. Typical domain organisations for each potential immune and immune-related gene were determined based on curated (reviewed) entries in uniprot (https://www.uniprot.org). Redundancy in the ORFs of interest, caused by using ORFs from both reference transcriptomes, was removed with cd-hit [139], using a sequence identity threshold of 1.0 at the protein level. In addition, where sequence alignments are shown or % sequence identities given, they have been carried out with Clustal Omega (https://www.ebi.ac.uk/Tools/msa/clustalo/).

## Supporting Information

S1 Fig Blast top-hit species distribution of a) combined and b) strand-specific *S*. *glomerata* ORF’s. Left graphs for both, a) and b) show the respective full-scale graphs, with the respective right graphs emphasising the fine scale features of the same graphs by adding a graph break.
(DOCX)

S2 Fig GO analysis of combined transcriptome ORF’s. GO-terms were determined with Blast2GO, using default parameters except for seq filter that was set to 50. Terms listed are based on a) biological process (Level 3), b) molecular function (Level 3) and c) cellular component (Level 4).
(DOCX)

S3 Fig GO analysis of strand-specific transcriptome ORF’s. GO-terms were determined with Blast2GO, using default parameters except for seq filter that was set to 50. Terms listed are based on a) biological process (Level 3), b) molecular function (Level 3) and c) cellular component (Level 4).
(DOCX)

S4 Fig Clustal Omega alignment of ECSIT sequences. Alignment of *S*. *glomerata* ECSIT transcript (c383034.graph_c0_seq1|m.66044) with ECSIT from *C*. *gigas* [GenBank:HQ225834] and *M*. *galloprovincialis* [GenBank:AHI17287].
(DOCX)

S5 Fig Clustal Omega alignment of big defensin sequences. Alignment of *S*. *glomerata* big defensin transcripts (c211420.graph_c0_seq1|m.6735, c349903.graph_c0_seq1|m.22175, c349903.graph_c0_seq2|m.22176, c359623.graph_c0_seq5|m.28084 and c354390.graph_c0_seq1|m.24530) with big defensins from *C*. *gigas* [GenBank:AEE92778, GenBank:AEE92768 and GenBank:AEE92775], *A*. *irradians* [GenBank:DQ334340] and *V*. *philippinarum* [GenBank:HM562672].
(DOCX)

S6 Fig Clustal Omega alignment of hydramacin. Alignment of *S*. *glomerata* hydramacin transcript (c328639.graph_c0_seq1|m.16116) with *V*. *philippinarum* hydramacin [GenBank:AGM14601].
(DOCX)

S1 File Detailed methods of experimental exposure trials. (DOCX)

S1 Table Mapping statistics. CLC Genomics Workbench (version 7.5) mapping statistics of individual tissue reads for determination of transcript distribution across the six tissues.
(DOCX)
