# Supplementary material for: Transcriptome Analysis of the Sydney Rock Oyster, Saccostrea glomerata: Insights into Molluscan Immunity
Source: PLoS One. 2016 Jun 3;11(6):e0156649. doi: 10.1371/journal.pone.0156649 (PMC4892480; doi:10.1371/journal.pone.0156649)
Supplement: S1 Fig — Left graphs for both, a) and b) show the respective full-scale graphs, with the respective right graphs emphasising the fine scale features of the same graphs by adding a graph break. (DOCX) [file pone.0156649.s001.docx]

**a)**

**b)**
