# Supplementary figures and images for: Transcriptome Analysis of the Sydney Rock Oyster, Saccostrea glomerata: Insights into Molluscan Immunity
Source: PLoS One. 2016 Jun 3;11(6):e0156649. doi: 10.1371/journal.pone.0156649 (PMC4892480; doi:10.1371/journal.pone.0156649)

**a)**


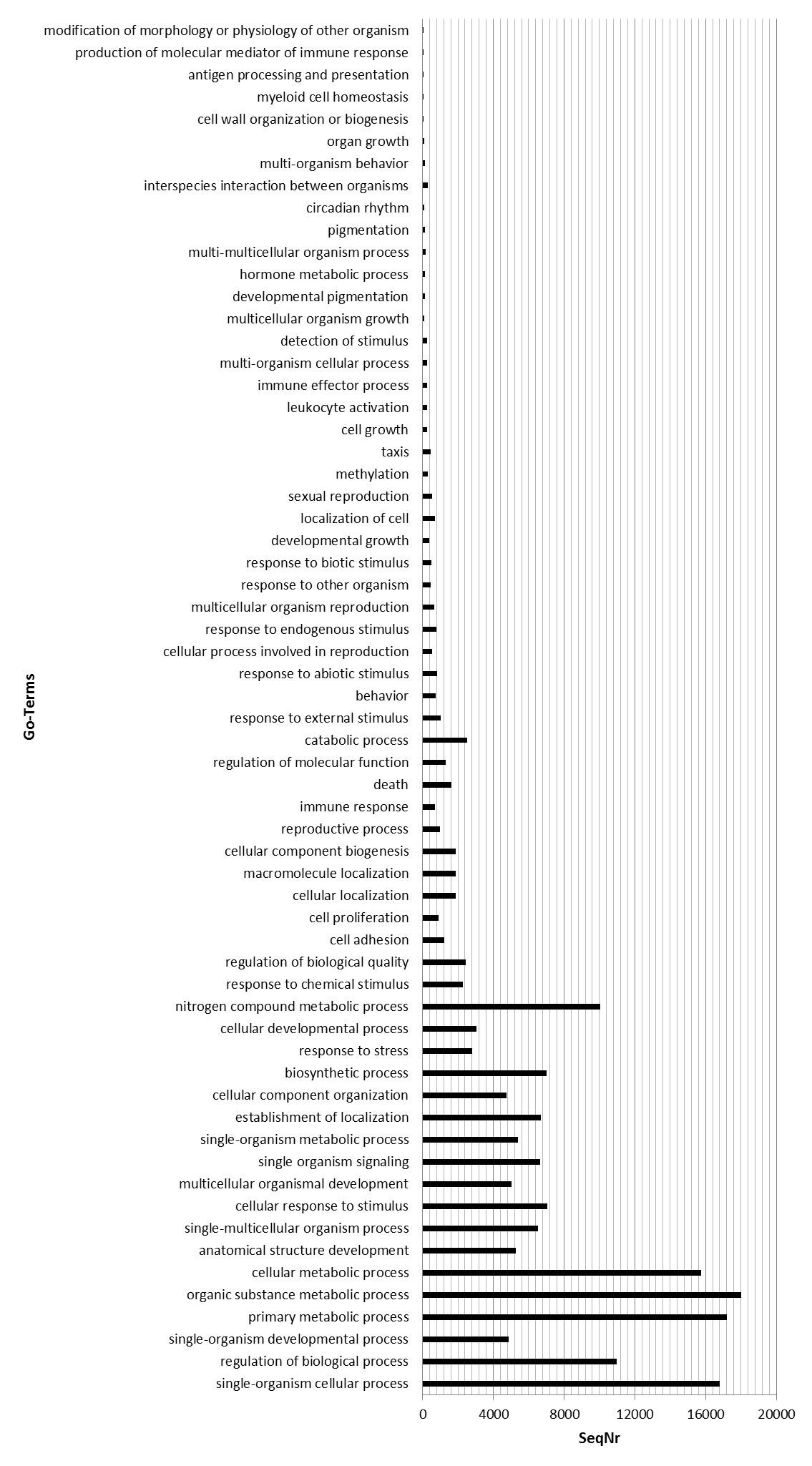


**b)**

**
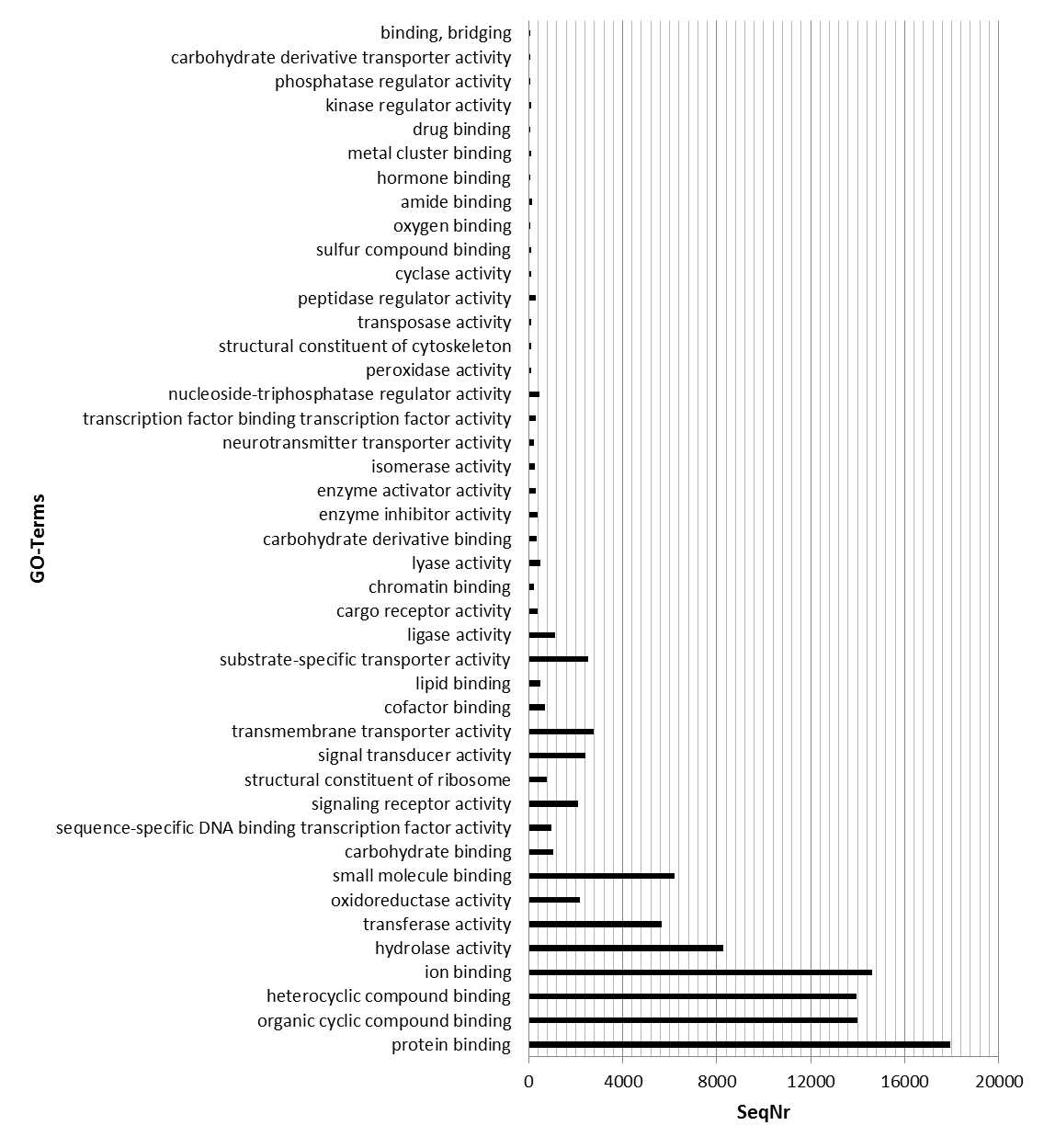
**

**c)**

**
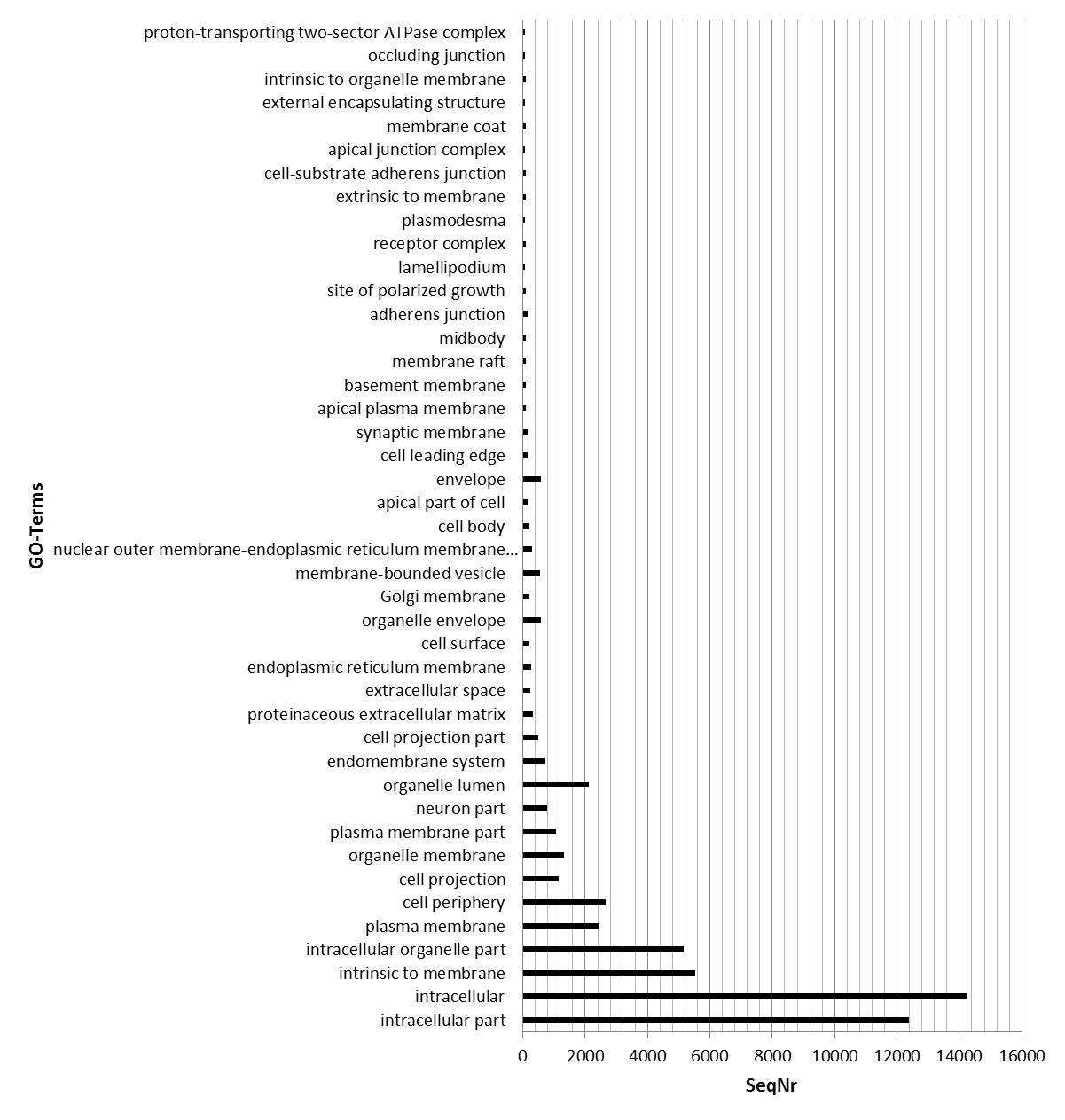
**

Supplement: S2 Fig — GO-terms were determined with Blast2GO, using default parameters except for seq filter that was set to 50. Terms listed are based on a) biological process (Level 3), b) molecular function (Level 3) and c) cellular component (Level 4). (DOCX) [file pone.0156649.s002.docx]

**a)**

**
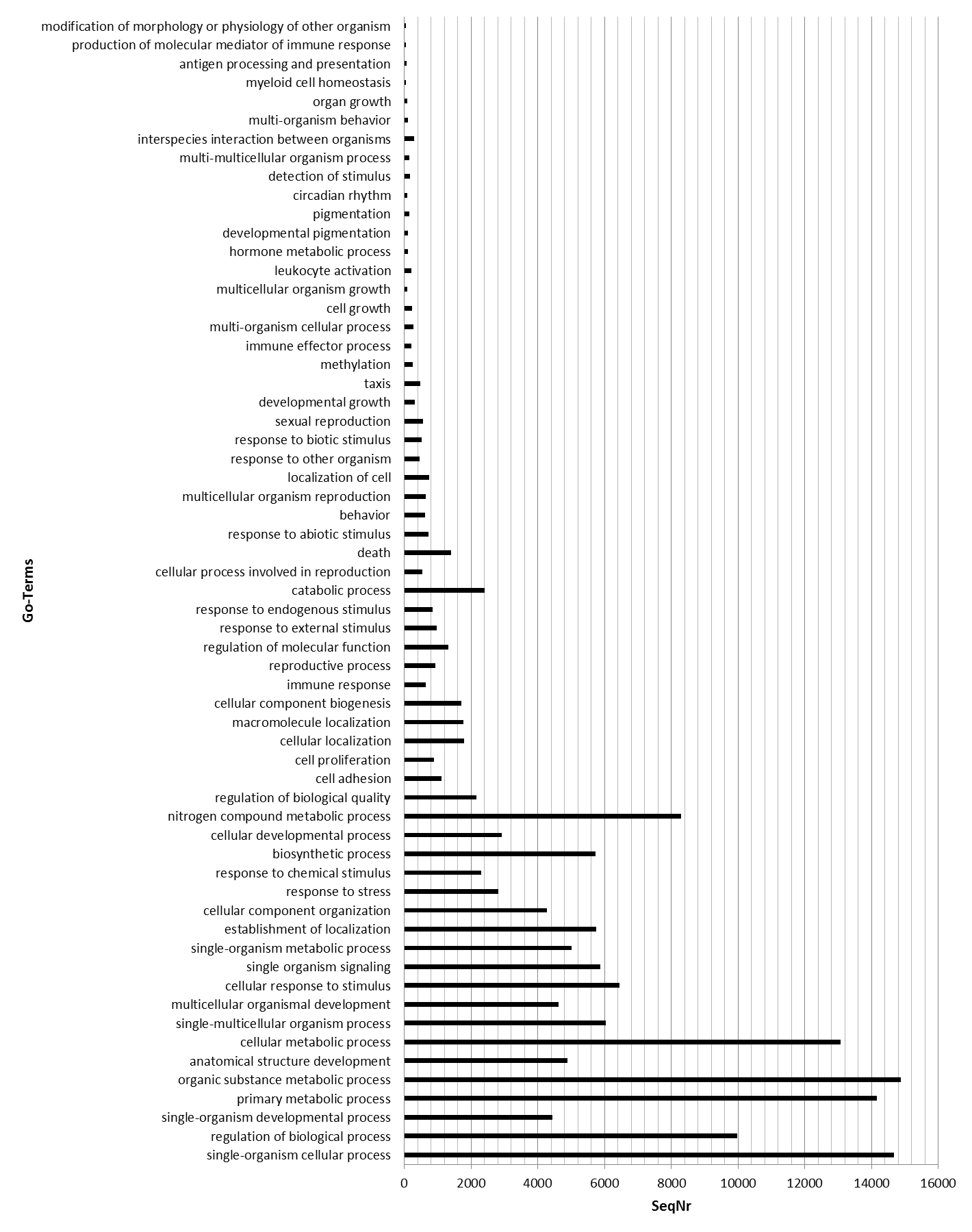
**

**b)**

**
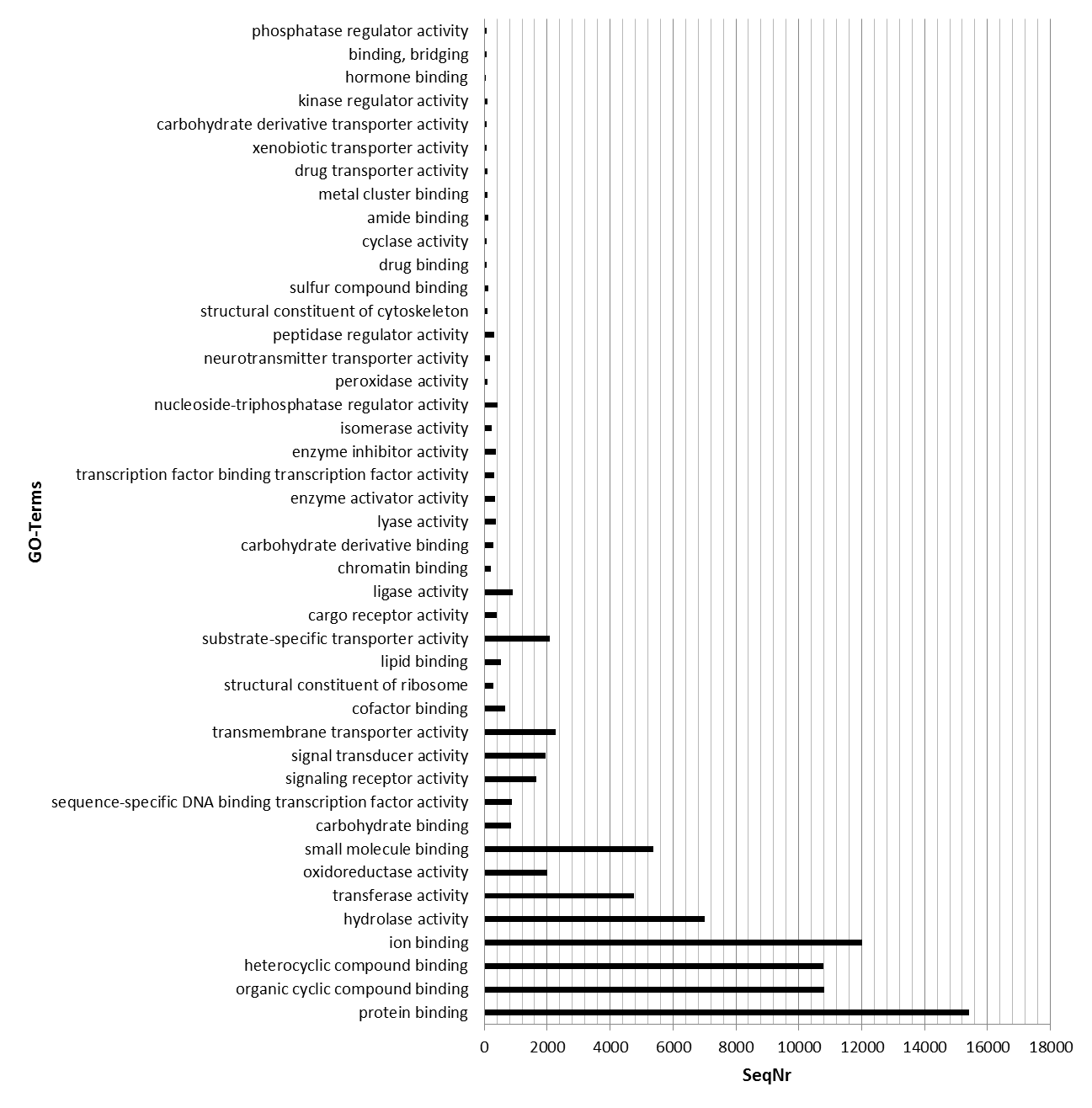
**

**c)**

**
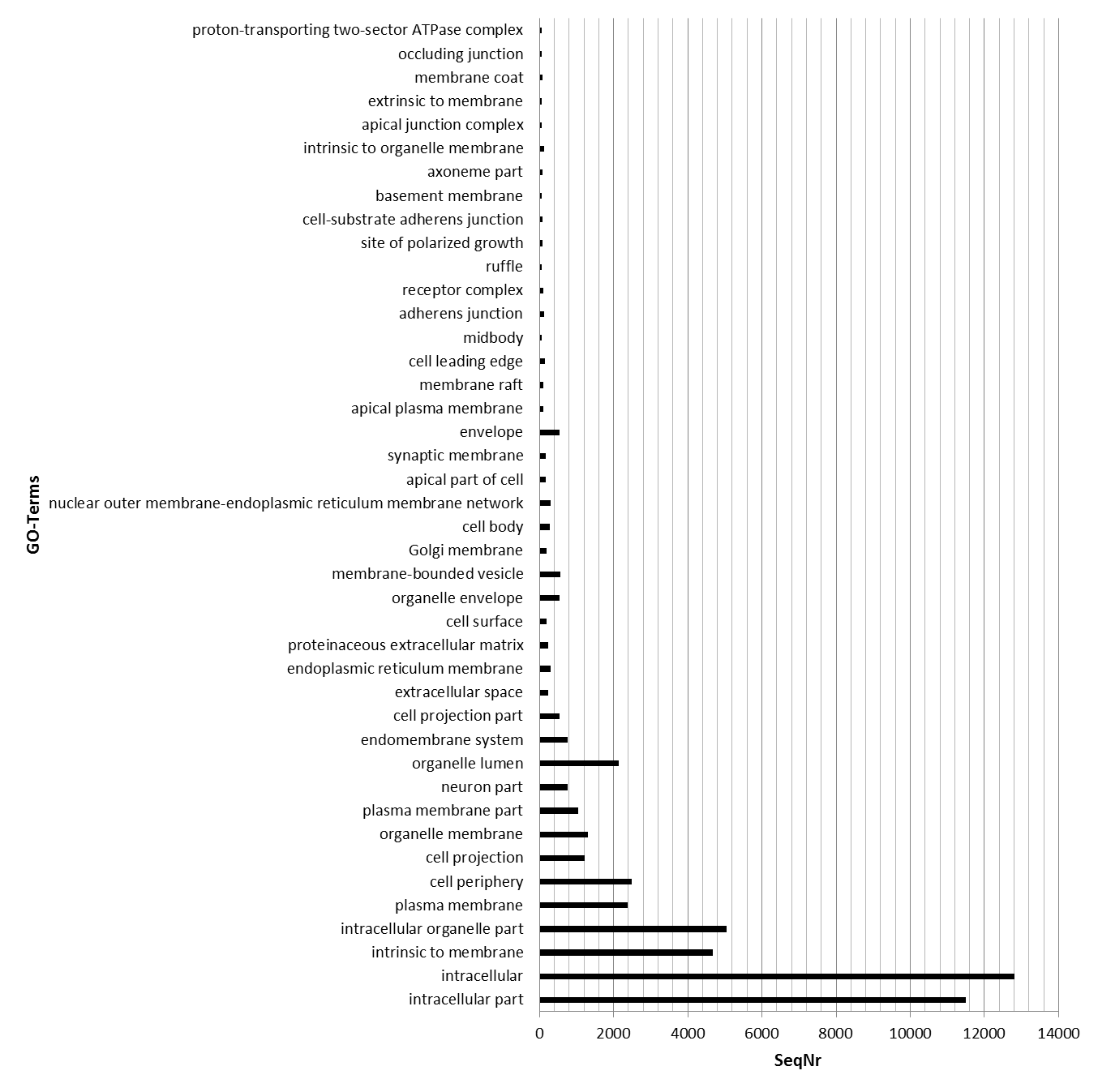
**

Supplement: S3 Fig — GO-terms were determined with Blast2GO, using default parameters except for seq filter that was set to 50. Terms listed are based on a) biological process (Level 3), b) molecular function (Level 3) and c) cellular component (Level 4). (DOCX) [file pone.0156649.s003.docx]
