# Supplementary material for: Transcriptome Analysis of the Sydney Rock Oyster, Saccostrea glomerata: Insights into Molluscan Immunity
Source: PLoS One. 2016 Jun 3;11(6):e0156649. doi: 10.1371/journal.pone.0156649 (PMC4892480; doi:10.1371/journal.pone.0156649)
Supplement: S4 Fig — Alignment of S. glomerata ECSIT transcript (c383034.graph_c0_seq1|m.66044) with ECSIT from C. gigas [GenBank:HQ225834] and M. galloprovincialis [GenBank:AHI17287]. (DOCX) [file pone.0156649.s004.docx]

*M.galloprovincialis* MLKITEPMQCIARLVIK-------RQ-KLLSSLYHSSVPSCKENKDIISTDKENIQIIKK

***S.glomerata* (m.66044)** -MTQRQILTCLRNLLSRDVGHAVKRKDLVANA-------SLLQNRNYHQTKSL-------

*C.gigas* -MTPRQTLLCLWNVLLRDARHVLVSDRILVQQASNKSIPQCSFVRTYHQTAPV-------

:. : : *: .:: : . : . . : .*

*M.galloprovincialis* ETDTGLMRPKFEASQDIQSPPPPEEDQPNPRDRKNKIDMRFIPKKY------TKTPQAIQ

***S.glomerata* (m.66044)** -----MFYNRFKNRKEVKKKSKED------EEQEKEIDAKLVEDKVKDLDVVAKIPYKIN

*C.gigas* -----MFYRRFLDRGKGKVKKKKK------KEEDQEIDAKIVEEELTQPEKVKKPGYKIR

:: :* . : . .:..::** ::: .: * *.

*M.galloprovincialis* ---FT----KKSALIFD-SLESKPKTLQTFKTACNIYLDKEGLYRRGHVEFIYAARDKLK

***S.glomerata* (m.66044)** VATFESKTDRFVNRIFNQVEQSGEKNQETFNTAVKIYKLNAGLYMRGHIEFGELALGKLE

*C.gigas* IPTYESTTDRYINRIFKKAEEQGEKTEETFNTAIKIYKLNAGLYVRGHIEFGDLALSKLE

: : **. :. *. :**:** :** : *** ***:** * **:

*M.galloprovincialis* EYNLNYDLNAYKALMNVFPKERLKPRSKIEAEFRRYPKQQDCAIEVLDTMGKNGVIPDVQ

***S.glomerata* (m.66044)** EYELQYNLEVYKMIFSIFPEGKYLPTSKIAAEFTPFPRQQDAALQILTKMFDNGVIPDDE

*C.gigas* EYELQYNLEVYKMIFSIFPEGKYLPKSKVDAEFIPFPRQQDAALRVLTKMFDNGVIPDEE

**:*:*:*:.** ::.:**: : * **: *** :*:***.*:.:* .* .****** :

*M.galloprovincialis* FYNLILSVFGEYSHVTRKLQRTMYWLPKFKHANPWPIPKLLPDNKIELAKLALKRMAFDI

***S.glomerata* (m.66044)** FGSMIISRFSQRSKVFTRFLRMLYWMPKFKYMNPWPIPRPPPKDPIQLAVLALKRMSFDL

*C.gigas* FGRMVISTFSQRSKVFTRFQRMLYWMPKFKHMNPWPVPRPPPKDPTQLAVMALKRMSFDL

* :::* *.: *:* :: * :**:****: ****:*: *.: :** :*****:**:

*M.galloprovincialis* NNELTVWKTTETEEN--PNEDTFIVSAQSAKQRELIKKLSPDKAVFVDGGYNVYLRNVMQ

***S.glomerata* (m.66044)** ETKVTVVNDSKEGETLSIRSGKFIASAQSPKQRELLSRHPSSKPIIVEGEHYVYLRKVCQ

*C.gigas* ETKITVVNDPRDS-----CSGKFIASAQSPKQIELLSKHPPSKPIIVEGEHYVYLRKVSQ

:.::** : . . .**.**** ** **:.: .* ::*:* : ****:* *

*M.galloprovincialis* TYFVLRADPEPEP---------EVKVDEEEDLFNWTTIFEEEKPSSIVLKKSVHEQEDGV

***S.glomerata* (m.66044)** KVFFLKTDPEGEITREDPDYYKALEEEDVEDMFDFTTPLSGEDQTALVPKKSVHEQEDGT

*C.gigas* KVFVLKTDREGELPGEEPDYYKAMEEEEDEEMFDFVTPLEEEEQTAIVPKKSVHEQEDGT

. *.*::* * * :: :: *::*::.* :. *. :::* **********.

*M.galloprovincialis* ILGMCITGSSTRDSLVSWIRYLQDSNPNLEHIPVVFTLKTVGQGTDVVKYDQQNQKQSSA

***S.glomerata* (m.66044)** VYAMCITSDSSKESLQDWVQFLQTTNSALEHIPVLFRIKDADSLLKETIRQEEEEEEL--

*C.gigas* VYGMCITSDSSKESVQDWIQFLQTNNPALEHIPVLFRIRDADFLLNETLKHEEEE-----

: .****..*:::*: .*:::** .* ******:* :: . . . .::::

*M.galloprovincialis* S

***S.glomerata* (m.66044)** -

*C.gigas* -
