# Supplementary material for: Transcriptome Analysis of the Sydney Rock Oyster, Saccostrea glomerata: Insights into Molluscan Immunity
Source: PLoS One. 2016 Jun 3;11(6):e0156649. doi: 10.1371/journal.pone.0156649 (PMC4892480; doi:10.1371/journal.pone.0156649)
Supplement: S5 Fig — Alignment of S. glomerata big defensin transcripts (c211420.graph_c0_seq1|m.6735, c349903.graph_c0_seq1|m.22175, c349903.graph_c0_seq2|m.22176, c359623.graph_c0_seq5|m.28084 and c354390.graph_c0_seq1|m.24530) with big defensins from C. gigas [GenBank:AEE92778, GenBank:AEE92768 and GenBank:AEE92775], A. irradians [GenBank:DQ334340] and V. philippinarum [GenBank:HM562672]. (DOCX) [file pone.0156649.s005.docx]

*V.philippinarum* ---------------MYKRTIF---IFYVFLVAMTTLSLCLDQKPEMEPFRKDAQQ-ALE

***S.glomerata* (m.6735)** ---------QLNASKMNQNKLYA--SFYVVLLTMFVPSTCLQVREGEEKLHRRNALAIGL

*A.irradians* ---------------MTRPSLVRCYSLFFTALIVMAIICP-AWSEEIPKSRKKRA----I

***S.glomerata* (m.22175)** QQLEGQNFSTVLALKMDKKTSLC--LLYIALLVLPAPILA-EVKTEKER---DRRQAIAL

***S.glomerata* (m.22176)** QQLEGQNFSTVLALKMDKKTSLC--LLYIALLVLPAPILA-EVKTEKER---ERRQAIAL

***S.glomerata* (m.28084)** ---------------MERNAFLF--ILYLTLLLAPVPILV-NARTEKKEPRNKRQAQVLL

***S.glomerata* (m.24530)** ---------------MEKKTFLC--VLYIILLVAPGPILA-RKAE-HEESRNKRQAPALL

*C.gigas*_3 (AEE92778) ---------------MERKSFLS--VFFIVLLVSPASILA-KTIKEEGETRNKRQAQILL

*C.gigas*_1 (AEE92768) ---------------MERKSFLS--AIFIVLLVSPASIMA-KTLEEVNETRNKRQAQALL

*C.gigas*_2 (AEE92775) ---------------MERKSFLS--AIFIVLLVSPASIMA-KTFEEVNDTRNKRQAQALL

* : ::. :

*V.philippinarum* -PSR----------------------------------------QRRWLHRRCL--SGRG

***S.glomerata* (m.6735)** PLLPELAGATVAPWLFGALALVYGYYALSNYRIKKGHDDTY----TCREYIHCA--ASKG

*A.irradians* -PI-AYVGMAVAPQVFRWLVRAYGAAAVTAAGVTLRRVINR---SRSNDNHSCY--GNRG

***S.glomerata* (m.22175)** -PLASLAGTAVAGPVFAALLAIYGAYTLAKYGIRV---------ISNRDSHPCG----RG

***S.glomerata* (m.22176)** -PLASLAGTAVAGPVFAALLAIYGAYTLAKYHI------------SDKDSHECG----RG

***S.glomerata* (m.28084)** -PIAIYAGIGVPPPVYLALVATYGSSVVASYAIRMSM-------FSDSDSHSCA--NNQG

***S.glomerata* (m.24530)** -PIAAYAGMTVSAPVFIALVAAYGLYQVTKYAIKKAKSTATTSSGTDYRNHACGGWFKDG

*C.gigas*_3 (AEE92778) -PIASYAGLTVTAPVFAALVAAYGIYAVTRYAIRKRR-----I-VMYSDSHSCA--NNRG

*C.gigas*_1 (AEE92768) -PIASYAGLTVSAPVFAALVTVYGAYALYRYNIRRRENSYQRI-RSDHDSHSCA--NNRG

*C.gigas*_2 (AEE92775) -PIASYAGLAVSPPVFAALVTAYGVYALYRYNIRRE--------NSDHDSHSCA--NNRG

* *

*V.philippinarum* FCRAICSIFEEPVRGNI-DCYFGYNCCRRMFSHYRTS

***S.glomerata* (m.6735)** LCKKSCNSKEVIDGGNS-VICGGEC------------

*A.irradians* WCRSSCRSYEYRG--GNLGVCGSYKCCVT--------

***S.glomerata* (m.22175)** YCRKKCQSHERVDWRLT-TGCGDYYCCV---------

***S.glomerata* (m.22176)** YCRKVCYKKEKIDWGLT-YECGNNYCCVYS-------

***S.glomerata* (m.28084)** WCKQRCFRTDRLSCDHS-ETCGRFYCCIPTT------

***S.glomerata* (m.24530)** RCRPRCLSGEYEDWRSS-DVCGGWKCCKWR-------

*C.gigas*_3 (AEE92778) WCRESCFSHEYTDWANTFGVCGSYFCCRPY-------

*C.gigas*_1 (AEE92768) WCRPTCFSHEYTDWFNN-DVCGSYRCCRPGRRG----

*C.gigas*_2 (AEE92775) WCRPTCYSYEYTDWFNN-DVCGSYRCCRPGRRG----

*: * :
