# Supplementary material for: Transcriptome Analysis of the Sydney Rock Oyster, Saccostrea glomerata: Insights into Molluscan Immunity
Source: PLoS One. 2016 Jun 3;11(6):e0156649. doi: 10.1371/journal.pone.0156649 (PMC4892480; doi:10.1371/journal.pone.0156649)
Supplement: S6 Fig — Alignment of S. glomerata hydramacin transcript (c328639.graph_c0_seq1|m.16116) with V. philippinarum hydramacin [GenBank:AGM14601]. (DOCX) [file pone.0156649.s006.docx]

***S.glomerata* (m.16116)** LQCSGRVLESVSSKGTNIRKLKMTAGKIILGATAMIFLLLVSNCPQTEGGLIGDCFDTWS

*V.philippinarum* ------------------------------MICSIVAILVMSTVFPRGDAFILECFETWS

::: :*::*. .:* :**:***

***S.glomerata* (m.16116)** RCTRWSYFLTGKAWLTCPQRCRCLGYNTGSCIKRNSKCPLTKKAYRCECTGRRIGPKPSK

*V.philippinarum* RCSGWSSGGTGWLWKSCQDRCEELGYSTGTCESADSNCRFVDKAYQCRCYGKTSGGSGSS

**: ** ** * :* :**. ***.**:* . :*:* :..***:*.* *: * . *.

***S.glomerata* (m.16116)** C------

*V.philippinarum* GGWWKRK
