# Supplementary material for: Transcriptome Analysis of the Sydney Rock Oyster, Saccostrea glomerata: Insights into Molluscan Immunity
Source: PLoS One. 2016 Jun 3;11(6):e0156649. doi: 10.1371/journal.pone.0156649 (PMC4892480; doi:10.1371/journal.pone.0156649)
Supplement: S1 File — (DOCX) [file pone.0156649.s007.docx]

# Experimental design

Tissues collected for analysis in this study were gill, mantle, adductor muscle, digestive system, gonad and haemolymph of *S. glomerata*. Individual haemolymph samples were always extracted first, using a 29 gauge x 0.5” sterile syringe with needle (Terumo, USA), and immediately frozen at -80°C. All other tissue samples were individually stored in RNAlater^TM^ (Ambion, Austin, TX) at 4°C overnight, and then frozen at -80°C until processing. Seawater (1 μm nominal filtered) used in all experiments was collected from Little Beach (Port Stephens, NSW, Australia). Acclimation period used in the experiments was based on a previous publication [1] and close observation of feeding as an indicator of acclimation, which had stabilised by the end of the acclimation period.

## 1. CO_2_ and temperature experiment

Wild, adult *S. glomerata* were collected from Cromarty Bay, Port Stephens (NSW, Australia) and randomly separated into two groups. Both groups were acclimated in a temperature controlled room for four days, after which nine oysters were randomly chosen from each group as controls and dissected. Acclimation parameters for the two groups were: a) 385 ppm (ambient) *p*CO_2_ and 22°C seawater and b) 385 ppm *p*CO_2_ and 28°C seawater. Following acclimation, oysters were randomly divided into three replicate 750 L header tanks per treatment, based on their acclimation temperature. Oysters acclimated to 22°C were either exposed to seawater with ambient *p*CO_2_ and temperature (22°C), or elevated *p*CO_2_ (1000 ppm) and ambient temperature, whereas animals acclimated to 28°C were exposed to either seawater of ambient *p*CO_2_ and elevated temperature (28°C), or elevated *p*CO_2_ and elevated temperature. Elevated temperature was maintained with a waterproof heater with thermostat for temperature control (± 0.5°C), and tank and CO_2_ delivery were set-up and maintained as previously described in Parker *et al.* [2]. During the acclimation period and exposure, oysters were fed with a mixture of Tahitian *Isochrysis* aff. *galbana* and *Chaetoceros muelleri* (2 x 10^9^ cells per oyster/day). In addition, complete water changes were carried out every second day, using seawater pre-adjusted to the respective *p*CO_2_ and temperature. After two and four weeks of exposure, nine oysters each per treatment (n=3 per tank) and sampling time point were collected.

## 2. Salinity and temperature experiment

Wild, adult *S. glomerata* were collected from Cromarty Bay, Port Stephens (NSW, Australia) and acclimated to seawater of ambient temperature (22°C) and salinity (33 ppt) in a temperature controlled room for four days, after which twelve oysters were randomly collected (control time point 0). The remaining oysters were divided into six treatment groups, with three replicate containers for each treatment (n=39 per treatment). Oysters were exposed to the following conditions for one week: a) ambient salinity and temperature, b) medium salinity (15 ppt) and ambient temperature, c) low salinity (10 ppt) and ambient temperature, d) ambient salinity and elevated temperature (30°C), medium salinity and elevated temperature and e) low salinity and elevated temperature. Levels of salinity used in this trial were based on a *S. glomerata* study by Nell and Dunkley [1]. Unchallenged oysters (reserve) were kept in 40 L trays inside a 750 L header tank with seawater of ambient salinity and temperature continuously recirculating through the trays. Throughout the experiment, reserve oysters were fed with a mixture of Tahitian *Isochrysis* aff. *galbana* and *Chaetoceros muelleri* at concentrations of 2 x 10^9^ cells per oyster/day, with the same diet given to the oysters during the initial acclimation period. All experimental oysters were held in 10 L plastic containers throughout the exposure period, with oxygen continuously bubbled into the water column. Waterproof heaters with thermostat for temperature control (± 0.5°C) were used to maintain a steady elevated temperature in the 30°C treatments. Experimental animals were fed once daily with 0.1 g of Nosan M-1 feed (Aquasonic, Australia) per oyster. This feed was provided to the experimental oysters to eliminate any potential impact salinity and temperature might have had on an algal diet. Feed was suspended in distilled water and had a particle size of 5 – 7.5 μm. Filtering and mortality were visually monitored and temperature and salinity measured twice a day. Mortalities during the experiment were recorded and replaced with the respective number of marked reserve oysters to maintain an equal number of oysters in each tank and treatment. The reserve oysters were not used in the transcriptome analysis. Full water changes were carried out every second day, using seawater pre-adjusted to its respective salinity and temperature. Nine samples (n=3 per container) were randomly collected after 1 h, two days and one week of exposure. Pre-trials had shown that *S. glomerata* opened their shells for filtering less than 5 min after being placed into ambient seawater. Therefore, for the 1 h trial only, oysters were considered to have been exposed for 1 h either a) 1 h after first opening their shells or b) if they did not open within 5 min, 1 h after that.

## 3. Polycyclic aromatic hydrocarbon (PAH) experiment

In this experiment, *S. glomerata* were exposed to two PAHs to mimic oil contamination. Pyrene and fluoranthene, two common components of oil contamination were chosen for this trial, with the concentration of both used based on levels recorded in a monitoring study carried out in the Port Stephens estuary (DPI, unpublished data). The following two levels of exposure were selected: a) concentration of both PAHs in the tested estuary (pyrene: 8.53 mg/kg, fluoranthene: 8.38 mg/kg) (medium dose), and b) concentration of total PAHs in the estuary (35.6 mg/kg) reconstructed with only pyrene and fluoranthene in their respective ratio (high dose). Rice flour with particle sizes of up to 50 μm was used as a carrier for the PAHs. Previous studies with *S. glomerata* had shown this species readily filtered and ingested rice flour from the water column and was therefore chosen as a carrier in this experiment [3].

### 3.1 Feed preparation and experimental set-up

All glassware used in the preparation of the feed was acid washed before use. One stock solution of pyrene and fluoranthene (Sigma-Aldrich, Australia) was prepared by dissolving 17.1 mg of pyrene and 16.7 mg of fluoranthene in 20 mL of dichloromethane (Honeywell, USA). To prepare spiked food for control, medium and high PAH treatments, 200 g of rice flour each were spiked with either 40 ml of dichloromethane (control) or with 40 mL of dichloromethane and the respective amount of stock solution for the medium and high PAH treatment. Once the flour had been spiked, it was dried in the fume-hood for four days, mixing gently in regular intervals throughout the drying time.

For the experimental exposure of the oysters, 30 glass containers with a holding capacity of 5 L were acid washed before use, along with hollow glass rods that were used to continuously bubble oxygen into the containers.

### 3.2 Oyster exposure

Wild, adult *S. glomerata* were collected from Cromarty Bay, Port Stephens (NSW, Australia) and acclimated to seawater of ambient temperature and salinity in a temperature controlled room for four days during which they were fed with a mixture of Tahitian *Isochrysis* aff. *galbana* and *Chaetoceros muelleri* (2 x 10^9^ cells per oyster/day). For the duration of the acclimation, oysters were kept in 40 L trays inside a 750 L header tank with seawater continuously recirculating through the trays. After acclimation, nine oysters were collected as control, then oysters were randomly split into the three treatment groups (control, medium and high PAH) and exposed to PAH for one week. All glass containers were filled with 4 L of ambient seawater and four oysters placed in each container. Each treatment group was replicated ten times, with the animals in the tenth container of each treatment used as a back-up in case of mortality. Each container was closed off with a lid that only had a single small opening at the top through which the glass rods were placed. Animals were fed with 20 mg/L/oyster of the respective spiked flour five times a day, with successful feeding and mortality visually observed. Complete water changes were carried out every second day using ambient seawater, with oysters and glass containers cleaned with seawater during each water change. At the conclusion of the experiment, a final complete water change was carried out after which all oysters were fed twice with control flour over a minimum of eight hour time period to allow for depuration of the digestive tracts of the experimental *S. glomerata*. Nine oysters (n=1 per container) were collected from each treatment 24 h after the start of the trial and at the end of the experiment after depuration.

## 4. Copper experiment

In this experiment, wild, adult *S. glomerata* (Cromarty Bay, Port Stephens, NSW, Australia) were randomly split into two treatments, control and exposed, with six replicate tanks for each treatment. Oysters in the exposed group were challenged with 50 μg/L of copper, in the form of copper(II)sulphate for six to seven weeks. During the exposure, control and treated oysters were kept in 200 L plastic tanks, with temperature (21°C), dissolved oxygen (6.7 mg/L), salinity (34.5 ppt) and pH (8.5) kept at a steady and optimal range for both treatment groups. Oysters were fed with a mixture of Tahitian *Isochrysis* aff. *galbana* and *Chaetoceros muelleri* (2 x 10^9^ cells per oyster/day), and complete water changes carried out every two days, using seawater. After each water change, 50 μg/L of copper was added to the water of the exposed oyster group to maintain a steady level of copper exposure throughout the trial. At the end of the trial, 12 oysters per treatment were randomly collected.

## 5. CO_2_ generational exposure experiment

For this experiment, wild and selectively breed (fast growth and QX disease resistance) *S. glomerata* oysters were exposed to elevated levels of *p*CO_2_ over multiple generations, with procurement of the oysters and the initial experimental set-up described in detail in Parker *et al.* [2]. In short, wild and selectively breed *S. glomerata* at the start of their reproductive conditioning were exposed to ambient (380 ppm) or elevated (856 ppm) *p*CO_2_ for five weeks. Gravid adult oysters were then strip spawned and the eggs fertilised with spermatozoa of the respective oyster and experimental group (i.e. eggs of wild ambient oysters fertilised with spermatozoa of wild ambient oysters). Fertilisation occurred under ambient and elevated *p*CO_2_ for all oyster and experimental groups, resulting in a total of eight larval experimental groups. After fertilisation, larvae were kept in their respective treatment (i.e. fertilised under ambient conditions were reared in ambient conditions) for 19 days, after which spat was brought back into their estuary for grow-out.

In this experiment, third generation adult wild and selectively breed oysters previously exposed (elevated) or not exposed (control) to 856 ppm *p*CO_2_ as larvae were again exposed to 856 ppm *p*CO_2_. Details of procurement, preparation and acclimation of oysters were as previously described [2]. After acclimation, the four oyster lines (wild control, wild elevated, selected control and selected elevated) were randomly divided across eight replicate 750 L header tanks of ambient seawater. Of these eight tanks, four were kept at ambient *p*CO_2_ and four at elevated *p*CO_2_, with one tank each maintained as a spare tank in case of mortalities. Each tank contained two flow-through 40 L trays that were split into two halves with each half carrying one group of oysters. This way all oyster lines could be exposed together in one tank. Oysters were fed a combination of Tahitian *Isochrysis* aff. *galbana* and *Chaetoceros muelleri* (2 x 10^9^ cells per oyster/day) and complete water changes carried out every second day. Oyster and tank maintenance as well as *p*CO_2_ exposure were carried out as previously described [2]. Nine oyster samples were collected from each of the four oyster lines at the end of the acclimation period and nine oysters (n=3 per tank) per oyster line and treatment (control and elevated) were collected after three weeks of *p*CO_2_ exposure.

# References

1. Nell JA, Dunkley PR. Effects of temperature, nutritional factors and salinity on the uptake of L-methionine by the Sydney rock oyster *Saccostrea commercialis*. Mar Biol. 1984;80(3):335-9.

2. Parker LM, Ross PM, O'Connor WA, Borysko L, Raftos DA, Pörtner H-O. Adult exposure influences offspring response to ocean acidification in oysters. Glob Chang Biol. 2012;18:82-92.

3. Wisely B, Reid BL. Experimental feeding of Sydney rock oysters (*Crassostrea commercialis* = *Saccostrea cucullata*): I. optimum particle sizes and concentrations. Aquaculture. 1978;15:319-31.
