# Supplementary material for: Transcriptome Analysis of the Sydney Rock Oyster, Saccostrea glomerata: Insights into Molluscan Immunity
Source: PLoS One. 2016 Jun 3;11(6):e0156649. doi: 10.1371/journal.pone.0156649 (PMC4892480; doi:10.1371/journal.pone.0156649)
Supplement: S1 Table — CLC Genomics Workbench (version 7.5) mapping statistics of individual tissue reads for determination of transcript distribution across the six tissues. (DOCX) [file pone.0156649.s008.docx]

|  |  | **% clean reads mapped** | | | | | |
| --- | --- | --- | --- | --- | --- | --- | --- |
| **Reference** | **type of read** | **hemolymph** | **gill** | **mantle** | **muscle** | **gonad** | **digestive** |
| Strand-specific | strand-specific | 90.97 | 90.14 | 91.44 | 94.19 | 92.11 | 92.89 |
| Combined | standard | 61.29 | 78.28 | 80.13 | 86.29 | 83.02 | 72.91 |
